# Ethical climate in the primary health care workplace: a mixed-method study*

**DOI:** 10.1590/1518-8345.7499.4756

**Published:** 2026-01-19

**Authors:** Lenna Eloisa Madureira Pereira, Laura Cavalcanti de Farias Brehmer, José Luís Guedes dos Santos, Graziele de Lima Dalmolin, Lucas Lorran Costa de Andrade, Flávia Regina Souza Ramos

**Affiliations:** 1 Universidade Federal do Pará, Departamento de Enfermagem, Belém, PA, Brazil. Universidade Federal do Pará Departamento de Enfermagem PA Belém Brazil; 2 Scholarship holder at the Conselho Nacional de Desenvolvimento Científico e Tecnológico (CNPq), Brazil. Scholarship holder at the Conselho Nacional de Desenvolvimento Científico e Tecnológico Brazil; 3 Universidade Federal da Santa Catarina, Departamento de Enfermagem, Florianópolis, SC, Brazil. Universidade Federal da Santa Catarina Departamento de Enfermagem SC Florianópolis Brazil; 4 Universidade Federal de Santa Maria, Departamento de Enfermagem, Santa Maria, RS, Brazil. Universidade Federal de Santa Maria Departamento de Enfermagem RS Santa Maria Brazil

**Keywords:** Primary Health Care, Ethics, Work, Surveys and Questionnaires, Nursing, Health Services

## Abstract

to analyze the ethical climate in the primary health care workplace in a municipality in the Amazon region, combining quantitative and qualitative data.

a convergent, parallel, mixed-method study. Qualitative data from interviews with 46 professionals underwent reflective thematic analysis. Quantitative data were obtained from 170 professionals using the Ethical Climate Inventory. Exploratory factor analysis tested the Ethical Climate Inventory model, proposing a new configuration for the items, with greater explanatory power than that maintained in the confirmatory factor analysis. Data integration was independent and presented in a joint display.

items with the highest factor loadings of the four factors of the Ethical Climate Inventory (1. principles and rules, 2. benevolence, 3. independence/individualism, and 4. sense of community/social responsibility) were analyzed in conjunction with the qualitative category, with multiple expressions of subjectivity and ethics in the workplace. No divergences were observed between the data, only convergences and complementarity in analytical syntheses by factor.

data integration broadened the understanding of the ethical climate in a little-explored scenario, demonstrating the distinction of this reality in terms of the constructs analyzed and the strength of ethical elements as a conceptual and analytical tool for workplace environments.

## Highlights

**(1)** Primary health care professionals have a cohesive ethical climate in their work environments. **(2)** Values vary across healthcare settings due to the diversity of care and technology. **(3)** Cooperation subverts the suffering imposed in adverse work environments.

## Introduction

The ethical climate (EC) was defined in the 1980s by Victor and Cullen as a set of behaviors, emotions, and perceptions that characterize an organization and are shaped by diverse elements, such as professional values, norms, concepts, and traditions. It was redefined in Nursing by Olson as the nursing team’s perceptions of how ethical issues are addressed or resolved in their specific work environment (WE), in this case, the hospital^(1)^. Such perceptions problematize organizational conditions to help workers in daily ethical reflections, involving care, management, policies, and decisions on ethical issues. Therefore, the EC promotes professional satisfaction and a healthy and safe environment for care, while a negative EC is a predictor of risks of harm to worker health^(2)^. Over the years, EC studies have been improving in both administration, psychology, and healthcare, although in this field, they have focused on hospital settings^(3)^ and their relationships with other factors, such as worker health^(2)^, decision-making skills, and interprofessional communication training^(4)^; nurse engagement^(5)^; moral distress^(6-7)^ and compassion fatigue^(7)^; intention to leave work^(8)^; job satisfaction^(1)^, among others^(3,9)^.

In the fields of nursing and hospitals, the pioneering study of the Hospital Ethical Climate Survey (HECS)^(9)^ stands out, authored by nurse Linda Olson. It is the most widely used, according to a review that analyzed 61 EC studies in 21 countries^(3)^. This same review demonstrated a gap in analyses in Primary Health Care (PHC) settings, observing variability only within hospital units, even when using general scales such as the Ethical Climate Questionnaire or ECIndex (ECQ/ECI). In addition to the most significant number of studies with nursing samples^(3)^, the ECQ has versions translated into different countries^(3,10-11)^; the HECS also has translations and applications on five continents, including Brazilian Portuguese^(3,9,12)^, the latter with the collaboration of the author of the original version. Also worth mentioning are the Social Capital and Ethical Climate at the Workplace Scale (SEW)^(13)^, which analyzes turnover intentions, injuries, and workplace accidents among professional caregivers; and the Ethical Decision-making Climate Questionnaire (EDMCQ)^(8)^, which assesses EC in intensive care units.

EC analysis has different approaches and theoretical frameworks, depending on the factors associated with the concept. Victor and Cullen’s EC typology was based on a 3x3 matrix that combines three ethical criteria (principle, benevolence, and egoism) with three loci of analysis (individual, local, and cosmopolitan)^(14)^, producing nine archetypes. According to this framework, in terms of ethical criteria, a CE encompasses benevolence when based on “concern for others/well-being of others”; principles and rules, when affected by the individual’s adherence to the norms and regulations of their profession and/or determined by the organization; and configured by independence/individualism (initially called “selfishness”), when actions are guided by personal beliefs and interests^(11,14)^. In terms of locus of analysis, the individual refers to personal interests; the local, when it concerns the interests of the system internal to the organization/groups; and cosmopolitan, when it involves the social system external to the organization. The graphic representation of the nine archetypes, according to Victor and Cullen’s original proposal, is shown in Figure 1. The term “selfishness” gave rise to the construct independence/instrumentalism and the term “principles” gave rise to the construct principles and rules.

**Figure 1-:** Locus of ethical analysis in decisions: individual, local, and cosmopolitan. Belém, PA, Brazil, 2025

		**Locus of analysis**		
		INDIVIDUAL	LOCAL	COSMOPOLITAN
**Ethical criteria**	SELFISHNESS	Self-interest	Company profits/interests	Efficiency
	BENEVOLENCE	Friendship	Team interest	Social responsibility
	PRINCIPLE	Personal morality	Organization rules and procedures	Professional codes and laws

Source: Ribeiro, et al, 2016^(15)^

Using this approach, Victor and Cullen developed a measurement instrument, the Ethical Climate Inventory (ECQ/ECI), adapted and validated in a Brazilian version^(15)^ for psychology and human resources management. Adaptations of the original version exist in foreign languages for the health and nursing fields, but not for Brazil^(1)^.

OA in healthcare differs according to cultural contexts, complexity and types of services, and teams involved, among others. In the hospital setting, a considerable number of studies address EC^(3,9,16-18)^. However, there are no instruments specifically designed to assess EC in PHC. This gap in the literature highlights the importance of linking the experiences of OA workers in PHC with the assessment of EC by workers in these settings.

In the Brazilian context, it is noteworthy that the country has a public and universal health system, which, despite advances, still faces challenges related to socioeconomic disparities, resource scarcity, service integration, and various barriers to access to health care^(19)^. Furthermore, Brazil has vast remote geographic areas, such as the Amazon, with limited service provision, a lack of health professionals, and adequate infrastructure. The scarcity of resources in these areas can result in long distances to be traveled for care and a lack of essential equipment and medications^(19)^.

The EC assessment has the potential to reveal how context-specific subjective and ethical conditions are perceived by workers in their impact on themselves, their work, and their care. Therefore, it is essential to direct this type of research to areas still underexplored in the health system, such as PHC services and those located outside of Brazil’s major urban centers. Due to the complexity of the phenomenon in question, a single-method approach proves limited in capturing its multiple dimensions. In this sense, the use of a mixed-methods research design is considered more appropriate, as it allows for the integration of different analytical perspectives, expanding the depth and scope of understanding of the object under investigation.

Thus, this study outlined three research questions linked to the mixed-methods design: the quantitative question sought to identify which factors or criteria assessed by the ECI are relevant in a PHC setting; the qualitative question investigated how the ethical dimension of PHC work is expressed from the perspective of workers; and the integrative question sought to understand the convergences and/or divergences between the results obtained through the ECI and workers’ reports on work-related work in PHC in a municipality in the Amazon region.

Given this scenario, the objective of this study was to analyze the ethical climate in the primary health care workplace in a municipality in the Amazon region, by combining quantitative and qualitative data. In other words, to explore convergences/divergences between the ECI assessment and the reports of OA by PHC workers.

## Method

### Study design

This is a convergent parallel mixed-methods study (QUANTI-QUALI), in which qualitative and quantitative data are collected concomitantly and with the same weighting. The mixed-methods approach was used because it allows the integration of quantitative and qualitative data, meeting the need to understand the phenomenon of EC s, the focus of this study, in a broader and more in-depth way^(20-21)^. The Mixed Methods Appraisal Tool (MMAT), version 2018^(22)^, was used as a methodological guide^(23)^.

### Study location and population

The study setting was the primary healthcare system (PHC) of a municipality in the metropolitan region of Belém, capital of the state of Pará, Brazil. Serving over 500,000 residents, the municipality under study has 12 primary healthcare units distributed across five regions. Two regions with seven municipal healthcare units were selected because they serve the largest populations. The study population comprised the total number of workers in the seven units, totaling 444.

### Quantitative study: sample, instrument, data collection and analysis

The quantitative phase was an analytical cross-sectional study that covered the entire population (workers from the seven units). The inclusion criteria were: tenured and/or contracted employees, with at least two months of service. Employees on vacation or leave, security guards, and residents of the units studied were excluded. Using a finite-population sample size calculation, considering a population of 444 workers with a sampling error of 5% and a 95% confidence level, we estimated a total of 169 participants.

Convenience sampling was used, with all workers who were present at the seven units on the days of data collection and those who had access via WhatsApp^®^ being invited to participate. A total of 214 workers were approached, of whom 44 declined, resulting in a sample of 170 participants from the following professions/positions across the seven units: nurses, nursing technicians and assistants, physicians, other higher-level professionals (social workers, psychologists, occupational therapists, nutritionists, and dentists), community health workers (CHW), other mid-level professionals (dental technicians and assistants, clinical pathology technicians, and radiology technicians), managers and administrative assistants, and general service agents. The terms “workers” or “employees” encompass the majority of participants with recognized professions and those who hold an occupation or position regardless of their educational qualifications, such as the last three mentioned.

The instrument used for data collection was the ECI (Ethical Climate Index), translated, adapted, and validated for the Brazilian context^(15)^. The original version, with 36 items, had already undergone revisions, including in Portuguese, so that the adopted instrument^(15)^ was validated in three constructs/factors and 19 items: benevolence (9 items), principles and rules (6 items), and independence and instrumentalism (4 items), maintaining consistency with the initial archetypes (Figure 1). The statistical tests for factor extraction were Kaiser and the principal axis method (PAF) with Promax rotation. Each item is measured on a 6-point Likert scale, from 1 (completely false) to 6 (completely true).

The instrument underwent no content change, but only an adjustment to one term in its questions – the term “company” was replaced by “health units”. Since the original instrument covers organizations in general and, in this specific application, the scenario referred only to PHC, this change was made to improve participants’ understanding, so that the other statements remained unchanged. Therefore, the decision was made not to perform further adaptation and validation, considering that its use could indicate such a future need. Authorization for use was obtained from the first author of the Brazilian version of the instrument^(15)^.

Data collection occurred after recruitment through in-person advertising at the units and via WhatsApp^®^, using contacts obtained through referrals from managers and workers. The instrument, after signing the informed consent form, was completed online, individually, from August 2020 to March 2021, via Google Forms. The questionnaire included a socio-occupational questionnaire (age, gender, marital status, children, marital status, postgraduate degree, and type and number of employment relationships), and the ECI^(14)^.

Statistical analysis was performed using the Statistical Package for the Social Sciences (SPSS) version 25 and IBM Amos Graphics v.20^(24)^. Initially, an analysis was carried out to verify the applicability of the scale to the studied scenario, using structural equations and confirmatory factor analysis (CFA) indices and, subsequently, exploratory factor analysis (EFA). The description of the results regarding the assessment of CE in this population was explored in detail in a previous publication^(25)^, including comparison tests between correlation analysis, when it came to relating ECI factors and categorical or quantitative variables.

In this study, Structural Equations Modeling (SEM) was used to test the ECI model in the context of PHC, considering the instrument’s structure and content. The fit indices and parameters used to evaluate the model, which proposed a new configuration for the items, with greater explanatory power than that maintained in the CFA, were: chi-square ratio per degrees of freedom (χ²/df) less than 3; (A)GFI representing the (Adjusted) Goodness of Fit greater than or equal to 0.95; RMSEA less than 0.06, where the 90% confidence interval for this indicator should not exceed 0.10; and Normed Fit Index (NFI), Comparative Fit Index (CFI), and Tucker-Lewis Index (TLI) greater than 0.9^(26-27)^.

EFA verification was also used to adapt the model to the scenario and population. The Kaiser-Meyer-Olkin (KMO) test was used to verify the sampling adequacy criterion (values equal to or less than 0.5 are considered inadequate for the factorial analysis) and Bartlett’s sphericity test (p<0.05) to evaluate the correlation matrix. Acceptable communality and factor loading values should be above 0.400, but for data integration in the mixed-method study, questions with loadings above 0.700 were considered^(28)^.

### Qualitative study: sample, instrument, data collection and analysis

The qualitative phase was exploratory and descriptive in nature and involved interviews with 46 PHC workers. Convenience sampling was adopted, following the same inclusion and exclusion criteria as the qualitative phase.

The instrument consisted of an interview guide consisting of seven open-ended questions, which was adjusted by three researchers and tested with two workers from the same services, one with a high school education and the other with a college degree, who were not included in the sample. The interview guide contained seven open-ended questions on: perception and elements of a healthy/positive environment; perception of an ethical environment; identification of conditions for a healthy/positive and ethical environment in their work setting; how to contribute to ethical environments; what situations are limiting and what can be improved to make environments ethical.

After signing the informed consent form, individual interviews were conducted in person by the first author between August and December 2020, subject to participant availability, and lasted an average of fifteen minutes. Data were recorded using the digital Voice Recorder^®^ Version 3 (42.0) application. The audio recordings were fully transcribed by the interviewer and organized using Atlas.ti software, whose features supported the qualitative analysis in the six phases of reflexive thematic analysis (RTA) (data familiarization; initial code generation; theme search; theme review; theme definition; report production)^(29-30)^. The entire process was double-checked until final categorization (first and last author). From the codebook, the category “Subjective and Ethical Elements,” with a magnitude (Mt) of 163 excerpts, was subsequently integrated with the quantitative data of the 19 ECI items.

Prior to integration, all qualitative data were coded and analyzed independently for integration. This analysis resulted in two large thematic networks grouping categories or code clusters, referring to: 1) Work Environment (WE) and Health, or ways of viewing the reality of one’s environment and designing desirable WE; this network gathered 322 excerpts/quotes (magnitude) and three categories (material, management and communication conditions with 10 codes in total); 2) Subjective and relational components of AT in PHC, composed of three categories with a total magnitude of 311 excerpts and 30 codes, dealing with the multiple expressions of ethics and subjectivity (ethical conceptions and questions, ethical problems and attributes for CE), work relationships and professional teams and perceptions and experiences of oneself at work (such as support, belonging, hope, but also disrespect and devaluation).

### Mixed study: data fusion procedures

After individual and independent analysis of the qualitative and quantitative data, the integration stage proceeded, which consists of producing a synchronous and consistent result greater than the sum of the qualitative and quantitative parts^(31)^.

Some statistical procedures were especially useful for defining results applicable to the integration or fusion of QUANTI-QUALI data, in terms of differences and similarities (convergent design)^(23)^. As previously mentioned, after verifying the ECI model in the PHC setting, using EFA, only the questions that had the greatest impact on their respective factors were considered, as assessed by the highest factor loading (>0.700).

For this mixed analysis, the questions with the highest factor loadings from the four ECI factors were associated with the 19 qualitative codes (subcategories) studied. A new analysis focused on 163 excerpts, initially contained in the multiple expressions of ethics and subjectivity category, now reviewed for their contributions to the deepening of relevant quantitative data. In other words, from the qualitative data set, only one group was created for this integration purpose, preserving the originality of qualitative data from other groups, which could be explored in exclusively qualitative publications.

Qualitative and quantitative data were integrated via joint display. Each association of ideas, convergent and/or divergent, according to the testimonies associated with the ECI, was inserted into domains. Thus, a weaving approach was created at the mixed-method level, or threads of encounters based on themes, or concepts, by concepts^(21)^.

### Ethical aspects

The research project received institutional approval from the Municipal Health Department and was approved by the Research Ethics Committee of the Federal University of Santa Catarina, under opinion No. 4079091, dated 06/09/2020, CAAE 32493920.4.0000.0121, in accordance with the Resolution of the National Health Council, No. 466/2012.

## Results

The results of the quantitative phase were reported in a previous publication^(25)^, and the data and interpretations, characteristic of a cross-sectional and correlational study, are not the subject of this article. For example, the correlation data are exclusive to that study, as are the average intensities for each item, maintaining the original three-factor scale configuration. This (mixed) study, however, presents a new four-factor configuration, obtained through complementary testing and as an exclusive contribution supported by the QUANTI-QUALI integration.

Indeed, the aforementioned interweaving, which explores threads of convergence between approaches around a theme/concept, goes beyond its mere summation and, therefore, is recommended to be reported in separate articles, no longer addressing the completeness of the QUANTI-QUALI results^(19)^. One of the advantages of mixed methods studies is the possibility of unfolding different academic products from a single project. In general, up to four distinct publications can be generated: one with the quantitative results, another with the qualitative results, a third with the integrated data analysis, and, finally, a methodological report highlighting the innovations in the study design and conduct. It is important to emphasize that this strategy does not constitute content repetition, but rather a legitimate and planned use of the database, in which each publication has its own identity, with specific analytical frameworks and objectives, respecting the ethical and scientific principles of the research^(19)^.

The participants in the quantitative phase were 170 healthcare workers, with an average age of 39 years (SD ± 10.2), mostly women (78.1%), with a partner (SD ± 49.4) and children (65.7%), with higher education (57.0%), civil servants working 40 hours per week (62.0%); and with no other employment relationships (64.1%).

The CFA parameters, considering the original ECI model for the studied scenario, presented the following values: chi-square (χ²) of 293.1 with 146 degrees of freedom (df), resulting in a χ²/df ratio of 2.00. The Goodness of Fit Index (GFI) was 0.854, and the Adjusted Goodness of Fit Index (AGFI) reached 0.810. The Normed Fit Index (NFI) registered 0.817, while the Tucker-Lewis Index (TLI) obtained 0.880. The Comparative Fit Index (CFI) was 0.897, and the Root Mean Square Error of Approximation (RMSEA) was 0.075, with a 90% confidence interval between 0.063 and 0.087.

The ratio between the chi-square statistic and degrees of freedom presented values within the expected range. The CFA parameters were very close to expectations, a result that suggested testing a new model using EFA in this study.

In the EFA, the indicators confirmed the adequacy of the ECI in four dimensions (Table 1), with a KMO value (above 0.8) suggesting acceptable sampling adequacy for EFA, as well as a significant Bartlett’s test of sphericity (p<0.01), indicating the factorability of the correlation matrix. Factor loading values ranged from 0.442 to 0.834, indicating that a moderate to substantial proportion of the variance in each item was shared with its respective factor.

The EFA, as shown in Table 1, presents the new configuration for the items: principles and rules factor (Factor 1 - PR), maintaining six items (questions 8, 10, 11, 12, 13, 15); benevolence factor (Factor 2 - BNV), with five items (1, 2, 3, 4, 6); independence/individualism factor (Factor 3 - IDP), with four items (16, 17, 18, 19); and the constitution of a new factor, called sense of community/social responsibility (Factor 4 - RS), with four items, which previously belonged to the benevolence factor (5, 7, 9, 14). Table 1 presents the 19 items of the original scale, distributed across the four factors, highlighting those that achieved preponderance (factor loadings above 0.700), which were used for integration with the qualitative data.

**Table 1-:** Exploratory factor analysis of the four factors of the Ethical Climate Inventory: principles and rules (PR*), benevolence (BNV^†^), independence/individualism (IDP^‡^), and sense of community/social responsibility (RS^§^). Belém, PA, Brazil, 2021

**Items**	**Components**			
	Factor 1 PR*	**Factor 2 BNV** ^†^	**Factor 3 IDP** ^‡^	**Factor 4 RS** ^§^
11. People are expected to comply, above all, with laws and professional standards.	**0.815**	0.314	-0.141	0.105
12. In this service, people are expected to strictly follow legal and professional standards.	**0.772**	0.265	-0.067	0.229
10. Everyone is expected to adhere to organizational standards and procedures.	**0.737**	0.273	-0.124	0.193
15. It is very important to strictly follow the rules and procedures of the healthcare facility.	**0.736**	0.143	-0.086	0.048
13. People are expected to always do what is best for clients and the public.	0.573	0.216	-0.276	0.343
8. Everyone is expected to be treated well when decisions are made.	0.512	0.331	-0.099	0.484
2. Our greatest concern is the well-being of everyone who works in this healthcare facility.	0.277	**0.834**	-0.013	0.173
1. The most important concern of the service is the well-being of everyone who works in it.	0.213	**0.753**	0.143	0.091
6. In the healthcare facility, our greatest concern is what is best for others.	0.343	0.663	-0.308	0.198
3. In the healthcare facility, people care about the well-being of each other.	0.367	0.488	-0.251	0.269
4. People care deeply about what is best for the staff.	0.281	0.442	0.057	0.440
17. In this service, people are guided by their own personal ethics.	0.087	-0.220	**0.783**	0.006
16. In this service, each person decides for themselves what they consider right and wrong.	-0.254	0.178	**0.717**	-0.066
18. In this service, people protect their own interests above all else.	-0.153	-0.126	**0.716**	-0.354
19. What matters in this service is what each person considers right or wrong.	-0.421	0.082	0.682	-0.125
14. People actively care about the interests of clients and the public.	0.115	0.094	-0.257	**0.742**
9. The people in this health unit have a strong sense of responsibility towards the external community.	0.229	0.352	-0.175	0.616
5. The first concern in this health unit is what is best for each individual.	0.083	-0.018	0.475	0.590
7. The people in this service place great importance on team spirit.	0.239	0.356	-0.384	0.560

*PR = Principles and rules; ^†^BNV =Benevolence; ^‡^IDP = Independence/individualism; ^§^RS = Sense of community/social responsibility

In the qualitative phase, 46 professionals were interviewed, with characteristics similar to those of the quantitative sample. They were also invited to participate in the quantitative phase or to provide information on new recruits.

The QUANTI-QUALI fusion point was the convergence of the 19 items of the ECI, which generated a new analytical process. The qualitative codes were grouped into: subjectivity or “sbj”; and ethics or “etc.” These are described below, in column 2 of Figures 3 and 4.

The multiple expressions of subjectivity (sbj) referred to the negative perceptions or effects that the TA produced in workers or that they considered limiting to a EC, for example, expressions of fear, devaluation and disrespect, negativity in envisioning changes, and perceiving themselves as unsupported in their needs. At the same time, and paradoxically, positive perceptions and effects were attributed to individual and team effort and proactivity, the support received, empathy, recognition, and satisfaction despite difficulties. all as elements of healthy AT and positive EC.

Expressions of ethics (etc.) were shown to be the basis or justification for professional attitudes and actions, sometimes based on personal ethics (values, principles, and family background) but primarily on professional ethics (principles and standards of a deontological nature, rights, and duties). Furthermore, moral doubts (insecurity and lack of support) and ethical conflicts (situations of moral disagreement between professionals or with users) were reported. While these hindered expressions of moral agency, they did not prevent the ability to act according to moral judgment and toward the promotion of CE.

**Figure 2-:**
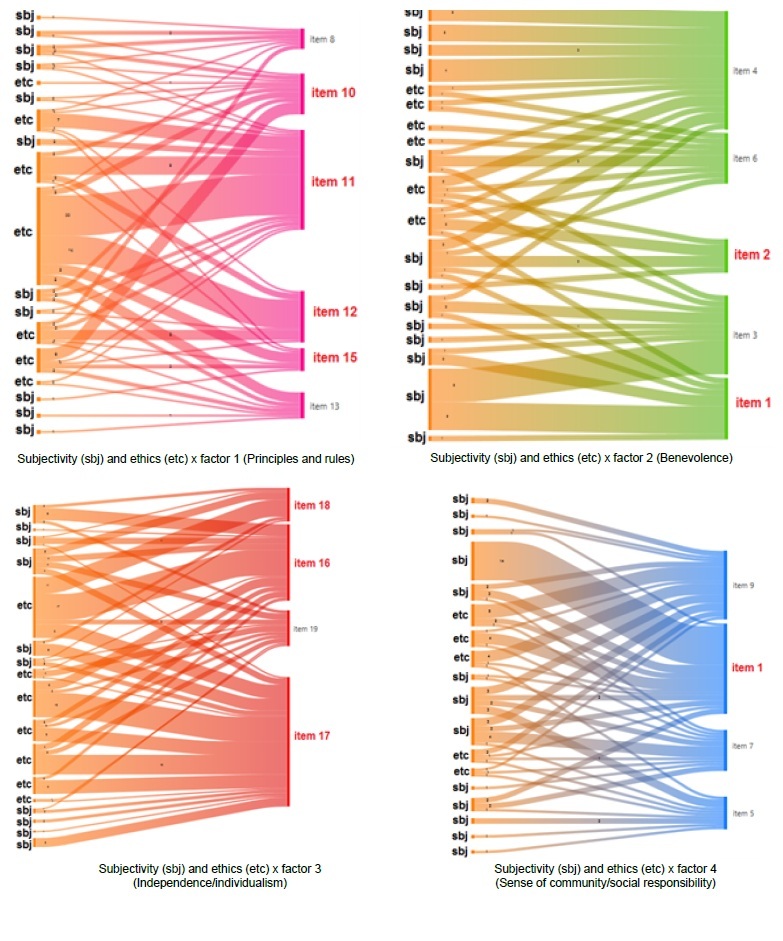
Co-occurrence analysis between qualitative codes and items of the 4 factors of the Ethical Climate Inventory. Belém, PA, Brazil, 2021

Figure 2 presents the analysis of the relationships (co-occurrence) of each of the four ECI factors with the qualitative codes of the thematic network “ethics and subjectivity”, obtained with the help of Atlas.ti^®^ software.

The integrated results are presented in two figures that highlight, for each scale factor, relevant summaries of the quantitative analysis and the convergences and divergences observed when articulated with subcategories from the qualitative phase, specifically addressing the expressions and importance of professional/personal ethics and perceptions and experiences in TA involving EC.

Only the items with the highest factor loadings (≥ 0.700) are described in columns, according to the EFA (Table 1), followed by the identification of the qualitative thematic category with a significant concentration of items (column 2) and examples of discursive excerpts that exemplify such convergent (+) or divergent (-) interpretation in column 3. As can be seen in Figure 2, there is co-occurrence with practically all categories, but the integration (Figure 3) explores the category with the greatest concentration of relationships.

The “principles and rules” factor (items 11 with 0.815; 12 with 0.772; 10 with 0.737; and 15 with 0.736) showed significant convergence with the “professional ethics” category (highest concentration), converging with discourses on the importance of professional and institutional codes, norms, and regulations for a positive ethical CE.

The benevolence factor (items 2 with 0.834; 1 with 0.753) converged with the perception of a healthy work environment, which highlights concern for the well-being of all those who work in the health unit or receive care there.

Figure 4 shows the independence and instrumentalism factor (items 17 with 0.783; 16 with 0.783; and 18 with 0.716), which included the qualitative code called moral agency. Convergence is observed when professionals’ ability and commitment to being guided by their own personal ethics while working are expressed. However, a complementary connotation was observed in the discourses—although consistent with the independence factor—also expresses the negative influence on the CE of individuals who are negligent, unaware, or poorly adhere to ethical standards in the workplace. This lack or negligence is revealed in the discourses as a factor in job dissatisfaction.

**Figure 3-:** Integration of results between Factor (1) Principles and rules (QUANTI^‡^) and Professional ethics category (QUALI^§^), and Factor (2) Benevolence (BNV^‡‡^) (QUANTI^‡^) and category Perceptions and experiences in the workplace (QUALI^§^). Belém, PA, Brazil, 2021

Factor 1 – Principles and rules (PR*)		
**ECI** ^†^ **– QUANTI** ^‡^	**Category QUALI** ^§^	**Convergences (+) or Divergence (-)**
Significant emphasis was placed on: • Laws and professional standards (expectation of compliance and strict adherence) (items 11 and 12); • Adherence to organizational standards and procedures (item 10); • Healthcare unit rules and procedures (strict adherence) (item 15).	Concentrated co-occurrence of items 11, 12 and 10 with the categories “Professional ethics”: values, principles and standards that guide professional practice and work in multidisciplinary teams, but which can generate ethical conflict when obligations are not fulfilled.	**(+):** *It’s about maintaining the confidentiality of things, of what happens here. Sometimes I comment on a particular patient, and I do so that it doesn’t get out of here, so that the person won’t tell anyone outside. It should stay within the team.* (CHW_05) ^||^ *This is unethical to me. If you entered the field as a professional, then you have to do what your field or profession requires.* (NURTECH_06) *I can’t interfere in another person’s professional conduct, a doctor’s, for example, because each has its own regulations, each has its own legislation regarding what they should do. So we have to work within the limits of our profession and practice.* (NUR_02)** **(-): not observed**
**Analytical synthesis:** The Ethical Climate in the APS Workplace ^††^ is related to professional ethics/professionalism, which, in turn, is related to both compliance with laws and professional standards and institutional norms and rules.		
**Factor 2 – Benevolence (BNV** ^‡‡^ **)**		
**ECI** ^†^ **– QUANTI** ^‡^	**Category QUALI** ^§^	**Convergences (+) or Divergence (-)** **(+):** *Everyone respects each other first and tries to do the best we can within the structure, from the work environment to the user.* (NUR_01)** *We are a family, we try to respect each other’s department. In terms of organization, we try to be as organized as possible.* (OAID_0) ^§§^ **(-): not observed**
Significant emphasis was placed on: • The well-being (benevolence) of all those who work in the healthcare unit (item 2); • The well-being (benevolence) of the people who work there (item 1).	Concentrated co-occurrence of items 2 and 1 with the subjective categories “Perceptions and experiences (constructed at the interface between positive and negative impressions), and Belonging to the group” (aggregating force capable of giving meaning to work)	
**Analytical synthesis:** The Ethical Climate in the APS Workplace ^††^ is related to benevolence, which, in turn, is expressed in workers’ perceptions and experiences of consideration for the common good and unity among professionals, as well as requiring and promoting relationships of mutual respect within the team..		

*PR = Principles and rules; ^†^ECI = Ethical Climate Inventory; ^‡^QUANTI = Quantitative; ^§^QUALI = Qualitative; ^||^CHW = Community Health Worker (+ the order of participation); ^¶^NURTECH = Nursing Technician (+ the order of participation); ^**^NUR = Nurse (+ the order of participation); ^††^APS = *Atenção Primária à Saúde*; ^‡‡^BNV = Benevolence; ^§§^OAID = Oral Aid (+ the order of participation)

**Figure 4-:** Integration of results between Factor (3) Independence/Instrumentalism (IDP^*^) (QUANT^‡^) and Personal Ethics category (QUAL^§^), and Factor Benevolence - Social Responsibility (QUANT^‡^) and Group Belonging and Ethical Climate category (QUAL^§^). Belém, PA, Brazil, 2021

Factor 3 – Independence and instrumentalism (IDP*)		
**ECI** ^†^ **– QUANTI** ^‡^	**Category QUALI** ^§^	**Convergences (+) or Divergence (-)**
Significant emphasis was placed on: • Guiding your own personal ethics (item 17); • Deciding for yourself what you believe is right and wrong (item 16); • Protecting your own interests (item 18)).	Concentrated co-occurrence of items 17, 16 and 18 with the ethical categories: “Moral agency (capacity to act to carry out fair actions in care – being a moral subject), personal ethics (personal values and attributes that influence action) and ethical conflict (dissents and clashes over values and moral judgments)”.	*I hear comments that I try to avoid. It only generates gossip when we give it free rein. Some people stop working and just talk and listen.* (TEC_NUR_03) ^||^ *I contribute with my attitudes, by example you can change many things.* (MED_03) ^¶^ *I try to do my ethical part with everyone, to see if we can have an ethical environment..* (CHW_12)** *The decisions of senior management are a bit unfair and this affects our working relationship.* (CHW_04)** *But there are situations where someone doesn’t know how to work and the person comes and points the finger in judgment.* (OAID_03) ^††^ *Talking about patient data, creating conflicts between colleagues. I think that’s a loss of ethics..* (NUR_03) ^‡‡^ **(-): not observed**
**Analytical Synthesis:** The Ethical Climate in the Primary Care Workplace ^§§^ is related to moral agency, as individuals exercise their moral judgment and define reasonable choices in situations involving moral problems or conflicts. Such judgments are based on facts and circumstances that, negatively (a complement that does not represent a divergence) or positively, refer to the ethical principles and values that serve as self-declared foundations (personal or professional).		
**Factor 4 – Sense of community/social responsibility (RS** ^||||^ **)**		
**ECI** ^†^ **– QUANTI** ^‡^	**Category QUALI** ^§^	**Convergences (+) or Divergence (-)**
Significant emphasis was placed on: • Concern for the interests of customers and the public.	Concentrated co-occurrence of items 14 with the category “subjectivity: Belonging to the group; and ethics: CE”.	**(+):** *I try to serve my clientele and help my colleagues as much as I can, within my limitations I help and contribute to the work.* (NUR_04) ^‡‡^ *I care a lot about the service and go beyond social service, I do what I can to make things happen..* (CHW_01)** *I can’t help patients as much as I’d like. Some patients can’t afford to pay for exams, so they charge me, and I try to talk to management. Sometimes they can’t schedule an appointment, so I feel bad, and it limits my work.* (CHW_05)** **(-): not observed**
**Analytical synthesis:** The Ethical Climate in the APS Workplace is also related to the sense of social responsibility of workers in the effort to do a decent job, which includes objective items that constitute a favorable work environment.		

*IDP = Independence and instrumentalism; ^†^ECI = Ethical Climate Inventory; ^‡^QUANTI = Quantitative; ^§^QUALI = Qualitative; ^||^NURTECH = Nursing Technician (+ order of participation); ^¶^MED = Medicine (+ order of participation); ^**^CHW = Community Health Worker (+ order of participation); ^††^OAID = Oral Assistant (+ order of participation); ^‡‡^NUR = Nurse (+ order of participation); ^§§^APS = Primary Health Care; ^||||^BAT-S = Secondary symptoms

Figure 4 also presents factor 4 - Social Responsibility (item 14 with 0.742), generated from a subdivision of the Benevolence factor (original scale). In convergence, the code “effort” expresses the active concern of professionals for the interests of the public and for PHC users; the criticism of the lack of physical and structural conditions that escape governability and limit the competence of workers, exposing the need for public policies in this TA.

## Discussion

### Differentiation of findings according to the ECI in Brazilian Portuguese

It was observed that the instrument used to measure CE in PHC settings is tailored to certain characteristics of these environments and is therefore not absolute. The translators of the Brazilian version themselves emphasize that future studies are needed to identify the suitability of the proposed framework and its predictive power of CE in different organizations^(15)^.

PHC professionals express consistent (stable) levels of CE in their work environments. However, based on the CE archetypes, where it is understood as an attribute of an organization that can be captured by individual perceptions^(15)^, it was possible to identify the following dominant factors in these environments: Principles and Rules; Benevolence; Independence (Individualism/Instrumentalism); and Social Responsibility (separated from the Benevolence factor).

A highlight of the study was the division of the Benevolence factor, generating the new Sense of Community/Social Responsibility factor. This represents a contribution to future research, since, although it does not express a conflict with the original author of the scale, when he situates the cosmopolitan locus of benevolence^(32)^, he already perceives EC as oriented toward the social system and collective interests. However, when observing a significant relevance of the sense of community/social responsibility, to the point of suggesting greater discrimination and constituting a new factor, a possible inadequacy of its approach as a simple locus of benevolence in the studied group may be glimpsed.

Studying EC in PHC requires further qualitative and quantitative analyses of the phenomenon, considering that the setting is immersed in complex and complementary subjective and objective dimensions. It is understood that the way in which meanings and understandings of ethics are attributed (its presence and/or absence in daily work) and the way ethics are “perceived and experienced” add meaning/value to the work environment for each professional.

Factor 1 – “Principles and Rules” had the highest factor loading among professionals. The statement corresponding to Item 11 of the ECI – “People are expected to comply, above all, with laws and professional standards” – generally shows that professionals understand and expect compliance with the relevant standards of each profession, which they understand as constituent elements of the CE present in these settings.

This finding was also similar in primary care, among nurses and physicians, who shared the perception of a favorable team climate when team goals are respected; and the recognition of nursing as a category closest to the ideals of the collective work process^(33)^.

Organizational ethics involve the consistency of actions with the organization’s mission, vision, and values, which necessitates dynamic forms of self-regulation, shared values among diverse stakeholders, and attention to their daily social interactions^(33)^. Values vary across healthcare settings, including due to the varying complexities of care or technology, and differ from the rules or norms that constitute the deontology of each profession^(34)^. Professional values and norms should be considered in their distinctions, but also in how they interact and integrate the formation of professional identities^(35)^.

This appropriation and local meaning given to norms suggests that ethical interventions in environments need not be limited to rules and regulations, but rather encouraged through ethical education for professionals, so that they can exercise honest and responsible judgment. Opening up dialogue between teams can help professionals better understand and modify the EC.

In Factor 2 – “Benevolence,” the statement “Our greatest concern is the well-being of everyone who works in this health unit” (item 2 of the ECI) may be related to recognition and satisfaction with managers and the team’s adherence to common goals. Cooperation among professionals and their involvement with users who seek PHC are elements reported as causes of job satisfaction among managers in the family health strategy^(36)^. This finding expresses how professional unity and a sense of teamwork reinforce common interests and are ingredients for maintaining a favorable work environment.

Cooperation is also seen as a means of subverting the suffering imposed by the adverse conditions in the OT. A sense of community can be equivalent to benevolence in the sense of contributing to the construction of common goals, mutual understanding, and respect for all who act under common standards^(37)^.

Factor 3 – “Independence and Individualism” expressed the sense of prioritizing self-interest within a given situation, or the interest in action determined by what the individual believes to be correct. The degree of adherence to the factor in the qualitative data was detected in the item “In this service, people are guided by their own personal ethics” (item 17 of the ECI).

Individualism can override the interests of the public or the team, contributing to work overload and excessive demands on coworkers. Excessive demands from oneself and others favor the occurrence of undesirable effects on both the environment and the public served, in addition to dissatisfaction on the part of the professional^(38)^. The same occurs in the exhaustion of relationships, where conflicting and ethically negative episodes can create an unfavorable climate and even moral distress^(39)^.

Obviously, dissatisfaction, work overload, burnout, and suffering are complex phenomena with multiple causes and consequences. It is only appropriate to point out the possible link between processes of exhaustion of cooperation and the encouragement of individualistic responses, the defense of personal interests, or self-preservation in such HTAs.

The problem of access to ethical education opportunities for workers is a reality in these environments. For CHW, there is still no code of ethics or entity responsible for overseeing their professional practice, much less the perception of ethical support from the professional category in the face of recurring situations in the care of PHC users. This gap does not prevent these workers from evaluating and formulating moral judgments about the reception and care environment^(40)^. Hence the urgent need for investment and the valorization of continuing health education (PHE) in the practice context of health services, with the inclusion of CHW in collective learning spaces^(41)^.

When there are no EPS incentives or actions related to situations requiring ethical competence, ethical actions/inactions go unnoticed, omitted, neglected, and, therefore, lack resolution. This aspect can cause moral distress to those who perceive such gaps. In these cases, it is generally the nurse who is sensitive to perceiving moral issues, as their training permeates such definitions and understandings^(42)^.

In Factor 4 – “Sense of Community/Social Responsibility,” the mixed-method study provided an additional contribution to a mutually complementary approach between Factors 2 and 4. Factor 4 is presented as a new component, derived from the second, not due to divergence, but rather due to deepening, since one possible interpretation is that the sense of community/team or social responsibility is presented as a way of achieving the common good (benevolence – Factor 2). At the same time, a form of internal benevolence (among peers) consolidates and integrates work teams so they can better fulfill their social role (a sense of community unfolding from benevolence). Social responsibility constitutes an ethical criterion, not merely a locus of analysis or a cosmopolitan characteristic of the CE^(15)^, as initially proposed by Victor and Cullen^(32)^. Thus, it is suggested that the simple cosmopolitan locus may be insufficient to explain the elements brought up by the mixed-method study, and may constitute a construct in itself.

Social responsibility is seen as an attribute of recognition and belonging to a public health service and system—of perceiving oneself as capable of guiding, assisting, and helping people seeking care. It is a notion appropriate for those who work with communities, nurture expectations of resolution, and face precariousness (structural, material, and safety) and powerlessness among these professionals, as they go beyond the limits of their unit or field of activity^(37)^.

Despite the richness of the findings, the study was limited by the lack of expanded sample stratification of some occupations that would have allowed for exploring differences in the factors involved. Furthermore, it is important to recognize that the period portrayed was highly impacted by the COVID-19 pandemic.

Future studies aimed at confirming the effectiveness of the ECI in other primary care settings in other regions of the country, with aspects different from what was found in the northern Amazon city, could provide new insights into CE typologies in healthcare TA. Although not intended as a methodological study, the application of the instrument suggests that it be adapted to the healthcare setting, including the social responsibility factor; and linguistic adjustments (e.g., using users instead of clients).

The study contributes to a deeper understanding of the concept of CE and its potential to support transformation in nursing care, especially in PHC. Capturing multiple data and perspectives is useful for supporting initiatives to improve nursing care for both workers and users. The quality of work outcomes and care delivery cannot be achieved without due attention to workers and the environments in which they operate, including the ethical dimensions of these environments. The study’s contribution to nursing is evident in that these workers are greatly affected by their nursing care and, also, by the leading role they play in transforming it, provided the intersection between ethics and nursing care is properly recognized and problematized. Furthermore, the mixed-method study was able to project an expanded interpretative configuration to the results obtained with the inventory, supporting the expression of the complexity of the object, thus providing indicative results for a future adaptation of the instrument for the health field.

## Conclusion

Several factors related to the CE assessment proved relevant in the analyzed PHC settings: the relevance of principles and rules, when referring to adherence to the standards relevant to each profession’s codes of ethics; benevolence, when referring to the well-being of the individuals working there as a guide for actions and choices; independence and instrumentalism, which raises the discussion of moral agents and the conflicts that can arise in the OT when ethical attitudes are neglected or ignored; and finally, the sense of community/social responsibility, derived from benevolence as a concern for providing good public service and fostering integrated collective work.

The integration of quantitative and qualitative data, with the four constructs/factors as a fusion point, broadened and deepened the understanding of the subject in analytical syntheses that: – reinforce the importance of professional ethics/professionalism, anchored in both professional and institutional norms; – the strength of benevolence directed both toward the community and toward relationships among professionals within the team; – the exercise of moral judgments, imposing challenges, but also the image of moral agency fundamental to their perception as moral subjects of actions; finally, an important distinction was made between the sense of community (among professionals) and social responsibility, as constituents of a CE in the studied setting. This distinction of the fourth construct was an important contribution and supported by quantitative and qualitative data, which is desirable in the application of validated instruments, which require support for their improvement.

The study brought to the discussion a little-explored setting in terms of CE: PHC and a municipality in the Amazon region. The object of study must necessarily consider the particularities of each environment – only then will it have the potential to transform scenarios, qualifying work experiences and their outcomes for the individuals and communities using them. The CE is a relevant conceptual and analytical tool for the ethical dimensions of environments and relationships in PHC, while mixed-method studies support the understanding of its complexity.

## Data Availability

The dataset for this article is available at the following links: https://www.dropbox.com/scl/fi/ghsc4rw2f73k8bayct5om/Relat-rio_21072021_FATOR1.pdf?rlkey=q8iw7p7uqsuewvuneddefc721&st=yw2jjg7c&dl=0 https://www.dropbox.com/scl/fi/szyv293q7vss8xsf3oywi/Relat-rio_21072021_FATOR2.pdf?rlkey=9anfauf9xnr2ml54pcvjaz0r8&st=fasfir3h&dl=0 https://www.dropbox.com/scl/fi/kb5wrpm1ar7ycapkjk03p/Relat-rio_21072021_FATOR3.pdf?rlkey=pjrxfroumomx47ls5gbhlj7go&st=ppk2ee2r&dl=0 https://www.dropbox.com/scl/fi/ffxf9ybpp442gwea406o0/Relat-rio_21072021_FATOR4.pdf?rlkey=g5t0pa17v91wa81c4brl6aicl&st=x3vr3d00&dl=0 https://drive.google.com/file/d/1mApBFHq7PF6qADF9mzkAhq2KDyuCQJwN/view?usp=drive_link
