# Supplementary material for: Ethical climate in the primary health care workplace: a mixed-method study
Source: Rev Lat Am Enfermagem. 2026 Jan 19;34:e4756. doi: 10.1590/1518-8345.7499.4756 (PMC12854651; doi:10.1590/1518-8345.7499.4756)
Supplement: Translation

# Translation: Clima ético no ambiente de trabalho na Atenção Primária à Saúde: um estudo misto*

**DOI:** 10.1590/1518-8345.7499.4757

**Authors:** Lenna Eloisa Madureira Pereira, Laura Cavalcanti de Farias Brehmer, José Luís Guedes dos Santos, Graziele de Lima Dalmolin, Lucas Lorran Costa de Andrade, Flávia Regina Souza Ramos

**Affiliations:** 1 Universidade Federal do Pará, Departamento de Enfermagem, Belém, PA, Brasil. Universidade Federal do Pará Departamento de Enfermagem PA Belém Brasil; 2 Bolsista do Conselho Nacional de Desenvolvimento Científico e Tecnológico (CNPq), Brasil. Bolsista do Conselho Nacional de Desenvolvimento Científico e Tecnológico Brasil; 3 Universidade Federal da Santa Catarina, Departamento de Enfermagem, Florianópolis, SC, Brasil. Universidade Federal da Santa Catarina Departamento de Enfermagem SC Florianópolis Brasil; 4 Universidade Federal de Santa Maria, Departamento de Enfermagem, Santa Maria, RS, Brasil. Universidade Federal de Santa Maria Departamento de Enfermagem RS Santa Maria Brasil

**Keywords:** Atenção Primária à Saúde, Ética, Trabalho, Inquéritos e Questionários, Enfermagem, Serviços de Saúde

## Abstract

analisar o clima ético no ambiente de trabalho da atenção primária à saúde em um município da região amazônica, por meio da articulação de dados quantitativos e qualitativos.

estudo misto paralelo convergente. Os dados qualitativos de entrevistas com 46 profissionais sofreram análise temática reflexiva. Já os dados quantitativos foram obtidos com 170 profissionais por meio do Inventário de Clima Ético. A análise fatorial exploratória testou o modelo do Inventário de Clima Ético, propondo nova configuração para os itens, com poder explicativo superior à mantida na análise fatorial confirmatória. A integração dos dados foi independente e apresentada em *joint display*

itens com maior carga fatorial dos quatro fatores do Inventário de Clima Ético (1. princípios e regras, 2. benevolência, 3. independência/individualismo, e 4. senso de comunidade/responsabilidade social) foram analisados em fusão à categoria qualitativa, com múltiplas expressões da subjetividade e ética no ambiente de trabalho, não sendo observadas divergências entre os dados, apenas convergências e complementação em sínteses analíticas por fator.

a integração dos dados ampliou a compreensão do clima ético em cenário pouco explorado, demonstrando a distinção dessa realidade em termos dos construtos analisados e a força dos elementos éticos em ferramenta conceitual e analítica dos ambientes de trabalho.

## Destaques

**(1)** Profissionais da Atenção Primária à Saúde apresentam clima ético coeso em seus ambientes de trabalho. **(2)** Valores variam em ambientes de saúde, pela diversidade assistencial e tecnológica. **(3)** Cooperação subverte o sofrimento imposto em ambientes de trabalho adversos.

## Introdução

O clima ético (CE) foi definido na década de 1980, por Victor e Cullen, como um conjunto de comportamentos, emoções e percepções que caracterizam uma organização e são moldados por elementos diversos, como valores profissionais, normas, concepções e tradições. Foi redefinido na Enfermagem, por Olson, como as percepções da equipe de enfermagem sobre como as questões éticas são abordadas ou resolvidas em seu ambiente de trabalho (AT) específico, no caso, o hospitalar^(1)^. Tais percepções problematizam as condições organizacionais para ajudar os trabalhadores nas reflexões éticas cotidianas, envolvendo a assistência, a gestão, as políticas e as decisões de problemas éticos; daí o CE promover a satisfação do profissional e o ambiente saudável e seguro para o cuidado, enquanto o CE negativo é um preditor para riscos de agravo à saúde do trabalhador^(2)^.

Ao longo dos anos, os estudos de CE foram se aprimorando tanto na área da administração, psicologia, quanto na área da saúde, embora neste campo tenha se concentrado em cenários hospitalares^(3)^ e em relações com outros fatores, como saúde do trabalhador^(2)^, habilidade de tomada de decisão e treinamento em comunicação interprofissional^(4)^; comprometimento de enfermeiras^(5)^; distresse moral^(6-7)^ e fadiga de compaixão^(7)^; intenção de abandonar o trabalho^(8)^; satisfação no trabalho^(1)^, entre outros^(3,9)^.

No campo da Enfermagem e da área hospitalar destaca-se o estudo pioneiro da escala *Hospital Ethical Climate Survey* (HECS)^(9)^, de autoria da enfermeira Linda Olson, o mais utilizado conforme revisão que analisou 61 estudos sobre CE em 21 países^(3)^. Esta mesma revisão demonstrou a lacuna de análises em cenários da Atenção Primária em Saúde (APS), ao observar variabilidade apenas em unidades hospitalares, mesmo quando usadas escalas gerais, como o *Ethical Climate Questionary* ou *ECIndex* (ECQ/ECI); além do mais expressivo número de estudos com amostras de Enfermeiros(as)^(3)^. O ECQ possui versões traduzidas para diferentes países^(3,10-11)^; o HECS também possui traduções e aplicações nos cinco continentes, incluindo o português brasileiro^(3,9,12)^, esta última com a colaboração da autora da versão original. Ainda vale citar o *Social Capital and Ethical Climate at the Workplace Scale* (SEW)^(13)^, que analisa as intenções de rotatividade, lesões e acidentes de trabalho entre cuidadores profissionais; e o *Ethical Decision-making Climate Questionnaire* (EDMCQ)^(8)^ que avalia o CE em unidades de tratamento intensivo.

A análise do CE possui diferentes abordagens e referenciais teóricos, de acordo com os fatores associados ao conceito. A tipologia do CE de Victor e Cullen foi baseada numa matriz 3x3 que combina três critérios éticos (princípio, benevolência e egoísmo) com três lócus de análise (individual, local e cosmopolita)^(14)^, produzindo nove arquétipos. Conforme esse referencial, em termos de critérios éticos, um CE contempla a benevolência quando se baseia na “preocupação com os outros/ bem-estar do outro”; os princípios e regras, quando é afetado pela adesão do indivíduo às normativas e regulamentos de sua profissão e/ou determinados pela organização; e configurado pela independência/individualismo (inicialmente denominada como “egoísmo”), quando o agir é orientado por crenças e interesses pessoais^(11,14)^. Em termos de lócus de análise, o individual se refere aos interesses pessoais; o local, quando recai sobre os interesses do sistema interno à organização/grupos; e cosmopolita, quando envolve o sistema social externo à organização. A representação gráfica dos nove arquétipos, conforme a proposta original de Victor e Cullen, consta na Figura 1, sendo que o termo “egoísmo” deu origem ao construto independência/instrumentalismo e o termo “princípios” originou o construto princípios e regras.

**Figura 1-:** Lócus de análise ética nas decisões: individual, local e cosmopolita. Belém, PA, Brasil, 2025

		**Lócus de análise**		
		INDIVIDUAL	LOCAL	COSMOPOLITA
**Critério ético**	EGOÍSMO	Auto interesse	Lucros/interesses da empresa	Eficiência
	BENEVOLÊNCIA	Amizade	Interesse da equipe	Responsabilidade social
	PRINCÍPIO	Moralidade pessoal	Regras e procedimentos da organização	Códigos profissionais e leis

Fonte: Ribeiro, et al, 2016^(15)^

A partir dessa abordagem, Victor e Cullen construíram um instrumento de medição, o Inventário de Clima Ético (ICE, em inglês ECQ/ECI), com adaptação e validação em versão brasileira^(15)^ no campo da psicologia e gestão de pessoas. Para o campo da saúde e Enfermagem existem adaptações da versão original em línguas estrangeiras, mas não para o Brasil^(1)^.

Os AT em saúde são diferenciados conforme contextos culturais, complexidade e tipos de serviços, equipes atuantes, dentre outros. No cenário hospitalar, há uma parcela considerável de estudos pertinentes ao CE^(3,9,16-18)^. Por outro lado, não existem instrumentos especificamente elaborados para avaliar CE na APS. Dessa lacuna identificada na literatura, justifica-se a importância de relacionar experiências dos trabalhadores em AT na APS com a avaliação de CE feita por trabalhadores desses cenários.

No cenário brasileiro, destaca-se o fato de o país contar com um sistema de saúde público e universal, o qual, apesar dos avanços, ainda enfrenta desafios relacionados a disparidades socioeconômicas, escassez de recursos, integração de serviços e diversas barreiras ao acesso à assistência médica^(19)^. Além disso, o Brasil conta com vastas áreas geográficas remotas, como na Amazônia, com oferta de serviços limitada, falta de profissionais de saúde e de infraestrutura adequada. A escassez de recursos nessas áreas pode resultar em longas distâncias a serem percorridas para obter atendimento e em falta de equipamentos e medicamentos essenciais^(19)^.

A avaliação do CE tem o potencial de revelar como condições subjetivas e éticas específicas de cada contexto são percebidas pelos trabalhadores em seus impactos sobre si mesmos, sobre o trabalho e sobre o cuidado. Diante disso, é fundamental direcionar esse tipo de investigação para campos ainda pouco explorados no sistema de saúde, como os serviços de APS e aqueles localizados fora dos grandes centros urbanos do Brasil. Em razão da complexidade que envolve o fenômeno em questão, uma abordagem unimétodo mostra-se limitada para captar suas múltiplas dimensões. Nesse sentido, o uso de um desenho de pesquisa de métodos mistos é considerado mais adequado, pois possibilita integrar diferentes perspectivas analíticas, ampliando a profundidade e o alcance da compreensão do objeto investigado.

Assim, delinearam-se, neste estudo, três questões de pesquisa articuladas ao desenho de métodos mistos: a questão quantitativa buscou identificar quais fatores ou critérios avaliados pelo ICE apresentam relevância em um cenário da APS; a questão qualitativa investigou como se expressa a dimensão ética dos AT da APS, segundo a perspectiva dos trabalhadores; e a questão integradora procurou compreender as convergências e/ou divergências entre os resultados obtidos por meio do ICE e os relatos dos trabalhadores sobre o AT na APS de um município da região amazônica.

Diante desse panorama, o objetivo deste estudo foi analisar o clima ético no ambiente de trabalho da atenção primária à saúde em um município da região amazônica, por meio da articulação de dados quantitativos e qualitativos. Ou seja, explorar convergências/divergências entre a avaliação feita por meio do ICE e os relatos sobre o AT dos trabalhadores da APS.

## Método

### Delineamento do estudo

Trata-se de um estudo misto paralelo convergente (QUANTI-QUALI), em que dados qualitativos e quantitativos são coletados concomitantemente e com mesma atribuição de peso. Utilizou-se a abordagem de métodos mistos por permitir a integração de dados quantitativos e qualitativos, atendendo à necessidade de compreender o fenômeno do CE, foco deste estudo, de forma mais ampla e aprofundada^(20-21)^. O *Mixed Methods Appraisal Tool*(MMAT), versão 2018^(22)^, foi utilizado como guia metodológico^(23)^.

### Local e população do estudo

O cenário do estudo foi a APS de um município pertencente à região metropolitana de Belém, capital do estado do Pará, Brasil. Para atender mais de 500 mil habitantes, o município estudado dispõe de 12 unidades básicas de saúde, distribuídas em cinco regiões. Foram selecionadas duas regiões que reúnem sete unidades municipais de saúde, por atenderem os maiores contingentes populacionais. A população do estudo compreendeu o número total de trabalhadores das sete unidades, perfazendo 444 trabalhadores.

### Estudo quantitativo: amostra, instrumento, coleta e análise dos dados

A fase quantitativa foi um estudo transversal analítico que abrangeu toda a população (trabalhadores das sete unidades). Os critérios de inclusão foram: servidores concursados e/ou contratados, com tempo de atuação de no mínimo dois meses. Foram excluídos servidores em férias ou licença, vigias e residentes das unidades pesquisadas. Por meio de cálculo amostral para população finita, considerando a população de 444 trabalhadores com erro amostral de 5% e nível de confiança de 95%, estimou-se 169 participantes.

Empregou-se uma amostragem por conveniência, sendo convidados a participar todos os trabalhadores que estavam presencialmente nas sete unidades nos dias de coleta de dados e, também, aqueles a quem se teve acesso via *WhatsApp*^®^. Foram abordados 214 trabalhadores, dos quais 44 declinaram, sendo a amostra constituída por 170 participantes, das seguintes profissões/cargos nas sete unidades: enfermeiros, técnico e auxiliares de enfermagem, médicos, demais profissionais de nível superior (assistente social, psicólogo, terapeuta ocupacional, nutricionista, dentista), agentes comunitários de saúde (ACS), demais profissionais de nível médio (técnico e auxiliar bucal, técnico em patologia clínica, técnico em radiologia), gerentes e assistentes administrativos, agentes de serviços gerais. Os termos trabalhadores ou servidores englobam a maioria dos participantes com profissões reconhecidas e os que exercem uma ocupação ou cargo independente da formação, como os últimos três citados.

O instrumento utilizado para coleta de dados foi o ICE traduzido, adaptado e validado para a realidade brasileira^(15)^. A versão original, com 36 itens, já tinha passado por revisões, inclusive em português, de forma que o instrumento adotado^(15)^ foi validado em três construtos/fatores e 19 itens: benevolência (9 itens), princípios e regras (6 itens) e independência e instrumentalismo (4 itens), mantendo coerência com os arquétipos iniciais (Figura 1). Os testes estatísticos para extração dos fatores foram Kaiser e método dos eixos principais (PAF) com rotação Promax. Cada item é medido por escala Likert de 6 pontos; do 1 (completamente falsa) ao 6 (completamente verdadeira). O instrumento não sofreu mudança de conteúdo, mas apenas ajuste de um termo em suas questões – o termo “empresa” foi substituído por “unidades de saúde”. Visto que o instrumento original contempla organizações no geral e, nesta aplicação em específico, o cenário se referia apenas à APS, tal alteração foi feita para melhor compreensão dos participantes, de modo que as demais afirmativas permaneceram inalteradas. Assim, a opção foi por não realizar nova adaptação e validação, até mesmo por considerar que seu uso poderia indicar tal necessidade futura. Foi obtida a autorização de uso junto a primeira autora da versão brasileira do instrumento^(15)^.

A coleta de dados ocorreu após recrutamento por publicização presencial nas unidades e via *WhatsApp*^®^ de contatos obtidos via indicação de gestores e trabalhadores. O preenchimento do instrumento, após assinatura do TCLE, foi *online*, individual, no período de agosto de 2020 a março de 2021, via formulário do *Google Forms*, contendo questionário sociolaboral (idade, gênero, estado civil, filhos, situação conjugal, pós-graduação e tipo e número de vínculos), e o ICE^(14)^.

A análise estatística foi realizada pelo *softwareStatistical Package for the Social Sciences* (SPSS) versão 25 e IBM *Amos Graphics* v.20^(24)^. Inicialmente, fez-se uma análise de verificação da aplicabilidade da escala ao cenário estudado, com o emprego de equações estruturais e índices de análise fatorial confirmatória (AFC) e, após, análise fatorial exploratória (AFE). A descrição dos resultados acerca da avaliação do CE nessa população foi detalhadamente explorada em publicação anterior^(25)^, incluindo testes de comparação entre análise de correlação, quando se tratou de relacionar fatores do ICE e variáveis categóricas ou quantitativas.

Neste estudo, foi usado o modelo de equações estruturais (*Structural Equations Modeling* – SEM) para testar o modelo do ICE no contexto da APS considerando a estrutura e conteúdo do instrumento. Os índices de ajustes e parâmetros para avaliar o modelo, e que propuseram uma nova configuração para os itens, com poder explicativo superior à mantida na AFC, foram: razão qui-quadrado por graus de liberdade (χ²/gl) menores que 3; (A)GFI representando o *(Adjusted) Goodness of Fit* maiores ou iguais a 0,95; RMSEA menores que 0,06 onde o valor do intervalo de confiança de 90% desse indicador não deve ultrapassar 0,10; valores de *Normed Fit Index* (NFI), *Comparative Fit Index* (CFI) e *Tucker-Lewis index* (TLI) maiores que 0,9^(26-27)^.

Empregou-se também, para adequar o modelo ao cenário e população, a verificação da AFE. Foram empregados os testes de Kaiser-Meyer-Olkin (KMO) para verificar o critério de adequação amostral (valores iguais ou menores de 0,5 são considerados inadequados para a fatorial) e teste de esfericidade de Bartlett (p<0,05) para avaliação da matriz de correlação. Os valores de comunalidade e carga fatorial aceitáveis devem estar acima de 0,400, porém para integração dos dados no estudo misto, foram consideradas as questões que apresentaram cargas acima de 0,700^(28)^.

### Estudo qualitativo: amostra, instrumento, coleta e análise dos dados

A fase qualitativa teve caráter exploratório-descritivo e foi realizada com entrevista de 46 trabalhadores da APS. Adotou-se amostragem de conveniência, seguindo os mesmos critérios de inclusão e exclusão da etapa qualitativa.

O instrumento foi um roteiro de entrevista composto por sete perguntas abertas, que foi submetido a ajustes por três pesquisadoras e testado com dois trabalhadores dos mesmos serviços, um com formação de nível médio e outro de nível superior, que não compuseram a amostra. O roteiro de entrevista continha sete questões abertas sobre: percepção e elementos de um ambiente saudável/positivo; percepção sobre ambiente ético; identificação de condições de um ambiente saudável/positivo e de um ambiente ético no seu cenário de trabalho; como contribuir para ambientes éticos; que situações são limitantes e o que pode ser melhorado para tornar os ambientes éticos.

Após assinatura do TCLE, as entrevistas individuais foram realizadas presencialmente, pela primeira autora, entre agosto a dezembro de 2020, conforme disponibilidade dos participantes e tiveram duração média de quinze minutos. O registro dos dados se deu via aplicativo digital Gravador de Voz^®^ Versão 3 (42.0). Os áudios foram integralmente transcritos pela entrevistadora e ordenadas no *software* Atlas.ti, cujas funcionalidades apoiaram a análise qualitativa, nas seis fases de análise temática reflexiva (ATR) (familiarização dos dados; geração de códigos iniciais; busca de temas; revisão de temas; definição de temas; produção do relatório)^(29-30)^. Todo o processo sofreu dupla conferência até a categorização final (primeira e última autora). Do livro de códigos (*codebook*) a categoria “Elementos subjetivos e éticos”, com magnitude (Mt) de 163 excertos, foi posteriormente integrada aos dados quantitativos dos 19 itens do ICE.

Previamente à integração, todos os dados qualitativos foram codificados e analisados de forma independente à finalidade de integrar. Desta análise derivaram duas grandes redes temáticas agrupando categorias ou agrupamento de códigos, referindo-se a: 1) Ambiente de trabalho (AT) e saúde, ou formas de ver a realidade do seu ambiente e projetar AT desejáveis; esta rede reuniu 322 excertos/citações (magnitude) e três categorias (condições materiais, gestionárias e de comunicação com 10 códigos no total); 2) Componentes subjetivos e relacionais do AT na APS, composta por três categorias com magnitude total de 311 excertos e 30 códigos, tratando das múltiplas expressões da ética e da subjetividade (concepções e questionamentos éticos, problemas éticos e atributos para o CE), das relações de trabalho e equipes profissionais e das percepções e experiências de si no trabalho (como apoio, pertencimento, esperança, mas também, desrespeito e desvalorização).

### Estudo misto: procedimentos de fusão dos dados

Após análise individual e independente dos dados qualitativos e quantitativos, procedeu-se à etapa da integração, que consiste em produzir um resultado síncrono e consistente maior que a soma de partes qualitativa e quantitativas^(31)^.

Alguns procedimentos estatísticos foram especialmente úteis para definir resultados aplicáveis à integração ou fusão de dados QUANTI-QUALI, em diferenças e semelhanças (*design*convergente)^(23)^. Como já citado, a partir da verificação do modelo do ICE no cenário da APS, a partir da AFE, foram consideradas apenas as questões que apresentaram maior impacto em seus respectivos fatores, avaliadas pela maior carga fatorial (>0,700).

Para o presente recorte misto, foram associadas as questões de maior carga fatorial dos quatro fatores do ICE com os 19 códigos (subcategorias) qualitativos estudadas. Uma nova análise recaiu sobre 163 excertos, inicialmente contidos na categoria múltiplas expressões da ética e subjetividade, agora revisados por suas contribuições ao aprofundamento de dados quantitativos relevantes. Ou seja, do conjunto de dados qualitativos, apenas um grupamento foi produzido para essa finalidade de integração, preservando a originalidade de dados qualitativos de outros grupamentos, passíveis de serem explorados em publicações exclusivamente qualitativas.

Os dados qualitativos e quantitativos foram integrados via *joint display.* Cada associação de ideias, convergentes e/ou divergentes, segundo os depoimentos associados ao ICE, foi inserida em domínios. Constituiu-se, assim, uma abordagem de tecelagem no nível do método misto, ou fios de encontros por base por tema, ou conceito por conceito^(21)^.

### Aspectos éticos

O projeto de pesquisa recebeu anuência institucional da Secretaria de Saúde do município e obteve aprovação pelo Comitê de Ética em Pesquisa da Universidade Federal de Santa Catarina, sob parecer nº 4079091, de 09/06/2020, CAAE 32493920.4.0000.0121, consonante à Resolução do Conselho Nacional de Saúde, nº466/2012.

## Resultados

Os resultados da etapa quantitativa foram relatados em publicação anterior^(25)^, sendo que nela os dados e interpretações, caraterísticos de um estudo transversal e correlacional, não são objeto do presente artigo. Por exemplo, os dados de correlação são exclusivos daquele recorte, assim como médias de intensidades por cada item, mantendo a configuração original da escala em três fatores. Já no presente recorte (misto) foi apresentada uma nova configuração, em quatro fatores, obtida em testes complementares e como contribuição exclusiva e suportada pela integração QUANTI-QUALI.

De fato, a já referida tecelagem, que explora fios de encontros entre as abordagens em torno de um tema/conceito, supera sua mera somatória e, por isso, é recomendado o relato em artigos distintos, não se tratando mais de abordar a completude dos resultados QUANTI-QUALI^(19)^. Uma das vantagens dos estudos de métodos mistos é a possibilidade de desdobrar diferentes produtos acadêmicos a partir de um único projeto. Em geral, podem ser geradas até quatro publicações distintas: uma com os resultados quantitativos, outra com os resultados qualitativos, uma terceira com a análise integrada dos dados e, por fim, um relato metodológico destacando as inovações na concepção e condução do estudo. Importante destacar que essa estratégia não se configura como repetição de conteúdo, mas uma utilização legítima e planejada do banco de dados, em que cada publicação possui identidade própria, com recortes analíticos e objetivos específicos, respeitando os princípios éticos e científicos da pesquisa^(19)^.

Os participantes da etapa quantitativa foram 170 trabalhadores de saúde, com idade média de 39 anos (DP± 10,2), majoritariamente mulheres (78,1%), com companheiro (DP± 49,4) e filhos (65,7%), com nível de formação superior (57,0%), concursados em regime de 40 horas semanais (62,0%); sem outros vínculos trabalhistas (64,1%).

Os parâmetros da AFC, considerando o modelo original do ICE para o cenário estudado, apresentaram os seguintes valores: qui-quadrado (χ²) de 293,1 com 146 graus de liberdade (df), resultando em uma razão χ²/df de 2,00. O *Goodness of Fit Index* (GFI) foi de 0,854, e o *Adjusted Goodness of Fit Index* (AGFI) alcançou 0,810. O *Normed Fit Index* (NFI) registrou 0,817, enquanto o *Tucker-Lewis Index* (TLI) obteve 0,880. O *Comparative Fit Index* (CFI) foi de 0,897, e o *Root Mean Square Error of Approximation* (RMSEA) apresentou valor de 0,075, com intervalo de confiança de 90% entre 0,063 e 0,087.

A razão entre a estatística qui-quadrado e graus de liberdade apresentou valores dentro do esperado. Os parâmetros da AFC se mostraram muito próximos do esperado, resultado que sugeriu testar novo modelo por AFE, no presente estudo.

Na AFE, os indicadores confirmaram a adequação do ICE em quatro dimensões (Tabela 1), com valor de KMO (acima de 0,8) sugerindo adequação amostral aceitável para AFE, bem como teste de esfericidade de Bartlett significativo (p<0,01) indicando a fatoribilidade da matriz de correlação. Os valores de carga fatorial variaram de 0,442 a 0,834 indicando que uma proporção moderada a substancial da variância em cada item foi compartilhada com seu respectivo fator.

A AFE, conforme Tabela 1, apresenta a nova configuração para os itens: fator princípios e regras (Fator 1 – PR), mantendo seis itens (questões 8,10, 11,12,13,15); fator benevolência (Fator 2 - BNV), com cinco itens (1, 2, 3, 4, 6); fator independência/individualismo (Fator 3 – IDP), com quatro itens (16,17,18,19); e a constituição de um novo fator, denominado senso de comunidade/responsabilidade social (Fator 4 – RS), com quatro itens, que antes pertenciam ao fator benevolência (5,7,9,14). A Tabela 1 apresenta os 19 itens da escala original, distribuídos nos quatro fatores, com destaque para aqueles que alcançaram preponderância (cargas fatoriais acima de 0,700), os quais foram utilizados para a integração aos dados qualitativos.

**Tabela 1-:** Análise fatorial exploratória dos quatro fatores do Inventário de Clima Ético: princípios e regras (PR*), benevolência (BNV^†)^, independência/individualismo (IDP^‡^) e senso de comunidade/responsabilidade social (RS^§^). Belém, PA, Brasil, 2021

Itens	Componentes			
	Fator 1 PR*	Fator 2 BNV ^†^	Fator 3 IDP ^‡^	Fator 4 RS ^§^
11.Espera-se que as pessoas cumpram, acima de tudo, as leis e as normas profissionais.	**0,815**	0,314	-0,141	0,105
12.Neste serviço, espera-se que as pessoas sigam estritamente as normas legais e profissionais.	**0,772**	0,265	-0,067	0,229
10.Espera-se aderência de todas as pessoas às normas e aos procedimentos organizacionais.	**0,737**	0,273	-0,124	0,193
15.É muito importante seguir estritamente as regras e procedimentos da unidade de saúde.	**0,736**	0,143	-0,086	0,048
13.Espera-se que as pessoas façam sempre o que é melhor para os clientes e o público.	0,573	0,216	-0,276	0,343
8.Espera-se que cada pessoa seja bem tratada quando as decisões são tomadas.	0,512	0,331	-0,099	0,484
2.A nossa maior preocupação é o bem de todos os que trabalham nesta unidade de saúde.	0,277	**0,834**	-0,013	0,173
1.A preocupação mais importante do serviço é o bem de todas as pessoas que nela trabalham.	0,213	**0,753**	0,143	0,091
6.Na unidade de saúde, a nossa maior preocupação concentra-se no que é melhor para a outra pessoa.	0,343	0,663	-0,308	0,198
3.Na unidade de saúde, as pessoas se preocupam com o bem de cada um dos outros.	0,367	0,488	-0,251	0,269
4.As pessoas preocupam-se muito com o que é globalmente melhor para os servidores.	0,281	0,442	0,057	0,440
17.Neste serviço, as pessoas guiam-se pela sua própria ética pessoal.	0,087	-0,220	**0,783**	0,006
16.Neste serviço, cada pessoa, por si própria, decide o que acha correto e incorreto.	-0,254	0,178	**0,717**	-0,066
18.Neste serviço, as pessoas protegem, acima de tudo, os seus próprios interesses.	-0,153	-0,126	**0,716**	-0,354
19.O que importa neste serviço é o que cada pessoa considera certo ou errado.	-0,421	0,082	0,682	-0,125
14.As pessoas preocupam-se ativamente com os interesses dos clientes e do público.	0,115	0,094	-0,257	**0,742**
9.As pessoas desta unidade de saúde têm um forte sentido de responsabilidade perante a comunidade exterior/externa.	0,229	0,352	-0,175	0,616
5.A primeira preocupação nesta unidade de saúde é o que é bom para cada indivíduo.	0,083	-0,018	0,475	0,590
7.As pessoas deste serviço dão muita importância ao espírito de equipe.	0,239	0,356	-0,384	0,560

*PR = Princípios e regras; ^†^BNV =Benevolência; ^‡^IDP = Independência/individualismo; ^§^RS = Senso de comunidade/responsabilidade social

Na etapa qualitativa, foram entrevistados 46 profissionais, com características distribuídas de forma semelhante à amostra quantitativa, sendo estes também convidados a participar da etapa quantitativa ou informar novos recrutáveis.

O ponto de fusão QUANTI-QUALI foram as convergências aos 19 itens da ICE, que geraram novo processo analítico. Os códigos qualitativos foram agrupados em: subjetividade ou “sbj”; e ética ou “etc”. Estes são descritos a seguir, na coluna 2 das Figuras 3 e 4.

As múltiplas expressões da subjetividade (sbj) se referiram às percepções ou efeitos negativos que o AT produzia nos trabalhadores ou que os mesmos consideravam limitantes de um CE, por exemplo, expressões de medo, desvalorização e desrespeito, negativismo nas formas de vislumbrar mudanças e perceber-se desassistido em suas necessidades. Ao mesmo tempo, e paradoxalmente, percepções e efeitos positivos eram atribuídos ao esforço e proatividade individual e de equipe, ao apoio recebido, a empatia, o reconhecimento e satisfação apesar das dificuldades; todos como elementos de AT saudável e CE positivo.

Já as expressões da ética (etc) se mostraram como a base ou justificativas das posturas e ações profissionais, ora baseada na ética pessoal (valores, princípios e formação familiar) mas, principalmente, na ética profissional (princípios e normas, de caráter deontológico, direitos e deveres). Além disso, foram reportadas dúvidas morais (insegurança e falta de sustentação), conflitos éticos (situações de desacordo moral entre profissionais ou com usuários), que apesar de dificultarem não impediam manifestações de agência moral, ou condições de agir conforme um julgamento moral e em direção à promoção do CE.

A Figura 2 apresenta a análise de relações (coocorrência) de cada um dos quatro fatores do ICE com os códigos qualitativos da rede temática “ética e subjetividade”, obtidas com o auxílio do *software* Atlas.ti^®^.

**Figura 2-:** Análise de coocorrência entre códigos qualitativos e itens do*s* 4 fatores do Inventário do Clima Ético. Belém, PA, Brasil, 2021

Os resultados integrados são apresentados em duas figuras que destacam, para cada fator da escala, sínteses relevantes da análise quantitativa e convergências e divergências observadas quando articulados com subcategorias da fase qualitativa, especificamente abordando expressões e importância da ética profissional/pessoal e percepções e experiências no AT envolvendo CE.

Apenas os itens com maiores valores de carga fatorial (≥ 0,700) são descritos em colunas, segundo a AFE (Tabela 1), seguidos pela identificação da categoria temática qualitativa com expressiva concentração de itens (coluna 2) e exemplos de excertos discursivos que exemplificam tal interpretação convergente (+) ou divergente (-) na coluna 3. Como se observa na Figura 2, há coocorrência com praticamente todas as categorias, mas a integração (Figura 3) explora a categoria com maior concentração de relações.

O fator “princípios e regras” (itens 11 com 0,815; 12 com 0,772; 10 com 0,737 e 15 com 0,736) mostrou expressivas convergências com a categoria “ética profissional” (maior concentração), convergindo com os discursos sobre a importância dos códigos, normativas e regulações profissionais e institucionais para um CE ético positivo.

**Figura 3-:** Integração de resultados entre Fator (1) Princípios e regras (QUANTI^‡^) e categoria Ética profissional (QUALI^§^), e Fator (2) Benevolência (BNV^‡‡^) (QUANTI^‡^) e categoria Percepções e experiências no ambiente de trabalho (QUALI^§^). Belém, PA, Brasil, 2021

Fator 1 – Princípios e regras (PR*)		
**ICE** ^†^ **– QUANTI** ^‡^	**Categoria QUALI** ^§^	**Convergências (+) ou Divergência (-)**
Evidenciada importante valorização de: Leis e normas profissionais (expectativa de cumprimento e estrito seguimento) (itens 11 e 12); Aderência às normas e procedimentos organizacionais (item 10); Regras e procedimentos da unidade de saúde (estrito seguimento) (item 15).	Coocorrência concentrada dos itens 11, 12 e 10 com as categorias “Ética profissional”: valores, princípios e normas que pautam o exercício profissional e o trabalho em equipes multiprofissionais, mas que podem gerar conflito ético quando obrigações são descumpridas.	**(+):** *É manter o sigilo das coisas, do que ocorre aqui. Às vezes comento sobre algum paciente em particular e comento para que não saia daqui que a pessoa não vá comentar com as pessoas lá fora. Que fique só entre a equipe.* (ACS_05) ^||^ *Isso pra mim não é ético, se você entrou na área como profissional então você tem que fazer o que a sua área ou profissão te cobra.* (TECENF_06) ^¶^ *Eu não posso interferir na conduta profissional de outra pessoa, do médico por exemplo, porque cada um tem a sua regulamentação, cada um tem a sua legislação do que deve fazer. Então a gente tem que trabalhar dentro dos limites que nossa profissão e atuação manda.* (ENF_02)** **(-): não observado**
**Síntese analítica:** O Clima Ético em Ambiente de Trabalho da APS ^††^ é relacionado à ética profissional/profissionalismo que, por sua vez, relaciona-se tanto ao cumprimento de leis e normas profissionais quanto à normas e regras institucionais.		
**Fator 2 – Benevolência (BNV** ^‡‡^ **)**		
**ICE** ^†^ **– QUANTI** ^‡^	**Categoria QUALI** ^§^	**Convergências (+) ou Divergência (-)**
Evidenciada importante valorização do: Bem (benevolência) de todos os que trabalham na unidade de saúde (item 2); Bem (benevolência) das pessoas que nela trabalham (item 1).	Coocorrência concentrada dos itens 2 e 1 com as categorias subjetivas “Percepções e experiências (construído na interface entre impressões positivas e negativas), e Pertencimento ao grupo” (força agregadora capaz de dar sentido ao trabalho)	**(+):** *Todos se respeitando primeiramente e tentando fazer o melhor que nós podemos dentro da estrutura, do ambiente de trabalho para o usuário.* (ENF_01)** *Nós somos uma família, a gente procurar respeitar o setor de cada um. Em termos de organização, a gente procura ser mais organizado possível.* (AUXBUC_0) ^§§^ **(-): não observado**
**Síntese analítica:** O Clima Ético em Ambiente de Trabalho da APS ^††^ é relacionado à benevolência que, por sua vez, se expressa nas percepções e experiências dos trabalhadores de consideração pelo bem comum e da união entre profissionais, assim como exige e promove relações de mútuo respeito na equipe.		

*PR = Princípios e regras; ^†^ICE = Inventário de Clima Ético; ^‡^QUANTI = Quantitativo; ^§^QUALI = Qualitativo; ^||^ACS = Agente Comunitário de Saúde (+ a ordem de participação); ^¶^TECENF = Técnico de Enfermagem (+ a ordem de participação); **ENF = Enfermeiro (+ a ordem de participação); ^††^APS = Atenção Primária à Saúde; ^‡‡^BNV = Benevolência; ^§§^AUXBUC = Auxiliar Bucal (+ a ordem de participação)

No fator benevolência (itens 2 com 0,834; 1 com 0,753) convergindo com a percepção do AT saudável que destaca a preocupação com o bem de todos os que trabalham na unidade de saúde ou ali são atendidos.

Na Figura 4, há o fator independência e instrumentalismo (itens 17 com 0,783; 16 com 0,783 e; 18 com 0,716), ao qual se integrou o código qualitativo denominado agência moral. Observa-se convergência quando se manifesta a capacidade e empenho dos profissionais por se guiarem pela sua própria ética pessoal enquanto trabalham. Entretanto, foi observada uma conotação complementar atribuída aos sentidos dos discursos – por mais que sejam coerentes com o fator independência – expressam também a influência negativa sobre o CE por parte de sujeitos que são negligentes, desconhecem ou pouco seguem os padrões éticos no AT. A falta ou negligência é desvelada nos discursos, como fator de insatisfação no serviço.

**Figura 4-:** Integração de resultados entre Fator (3) Independência/instrumentalismo (IDP*) (QUANT^‡^) e categoria Ética pessoal (QUAL^§^), e Fator Benevolência - Responsabilidade social (QUANT^‡^) e categoria Pertencimento ao grupo e Clima Ético (QUAL^§^). Belém, PA, Brasil, 2021

Fator 3 – Independência e instrumentalismo (IDP*)		
**ICE** ^†^ **– QUANTI** ^‡^	**Categoria QUALI** ^§^	**Convergências (+) ou Divergência (-)**
Evidenciada importante valorização de: Guia da sua própria ética pessoal (item 17); Por si própria decidir o que acha correto e incorreto (item 16); Proteção dos próprios interesses (item 18).	Coocorrência concentrada dos itens 17, 16 e 18 com a categorias éticas: “Agência moral (capacidade de agir para concretizar ações justas no cuidado – ser sujeito moral), ética pessoal (valores e atributos pessoais que influenciam o agir) e conflito ético (dissensos e embates por valores e juízos morais)”.	*Eu ouço comentários que procuro até fugir. Só gera fofoca quando a gente dá trela. Tem gente que deixa de trabalhar e fica falando, escutando.* (TEC_ENF_03) ^||^ *Contribuo com as minhas atitudes, pelo exemplo você consegue modificar muitas coisas.* (MED_03) ^¶^ *Eu tento fazer a minha parte ética com todas as pessoas, para ver se conseguimos ter um ambiente ético.* (ACS_12)** *Das decisões da alta gerência, elas são um pouco injustas e isso afeta a nossa relação no trabalho.* (ACS_04)** *Mas tem situações em que um não sabe como se trabalha e a pessoa vem e aponta o dedo julgando.* (AUXBUC_03) ^††^ *Ficar falando os dados de pacientes, ficar com conflito entre os colegas. Eu acho que aí já perde a ética.* (ENF_03) ^‡‡^ **(-): não observado**
**Síntese analítica:** O Clima Ético em Ambiente de Trabalho da APS ^§§^ é relacionado à agência moral, na medida em que os sujeitos exercitam seu julgamento moral e definem escolhas razoáveis diante de situações que envolvam problemas ou conflitos morais. Tais julgamentos recaem sobre fatos e circunstâncias que, negativa (complemento que não representa uma divergência) ou positivamente, remetem aqueles princípios e valores éticos que servem de base autodeclaradas (pessoais ou profissionais).		
**Fator 4 – Senso de comunidade/responsabilidade social (RS** ^||||^ **)**		
**ICE** ^†^ **– QUANTI** ^‡^	**Categoria QUALI** ^§^	**Convergências (+) ou Divergência (-)**
Evidenciada importante valorização da: Preocupação com os interesses dos clientes e do público.	Coocorrência concentrada dos itens 14 com a categoria “subjetividade: Pertencimento ao grupo; e ética: CE”.	**(+):** *Eu procuro atender a minha clientela e ajudo meus colegas no que eu posso, no que está dentro da minha limitação eu ajudo e contribuo para o trabalho.* (ENF_04) ^‡‡^ *Eu zelo muito pelo serviço e vou além do serviço social, faço o que posso para as coisas acontecerem.* (ACS_01)** *Eu não posso ajudar tanto o paciente quanto eu gostaria. Tem paciente* *que não tem condições de pagar por exames, eles me cobram e eu tento falar com a gerência, às vezes não conseguem marcar então eu me sinto mal e limita meu trabalho.* (ACS_05)** **(-): não observado**
**Síntese analítica:** O Clima Ético em Ambiente de Trabalho da APS é também relacionado ao senso de responsabilidade social dos trabalhadores no esforço de fazer um trabalho digno, o que inclui itens objetivos constituinte de um ambiente favorável de trabalho.		

*IDP = Independência e instrumentalismo; ^†^ICE = Inventário de Clima Ético; ^‡^QUANTI = Quantitativo; ^§^QUALI = Qualitativo; ^||^TECENF = Técnico de Enfermagem (+ a ordem de participação); ^¶^MED = Medicina (+ a ordem de participação); **ACS = Agente Comunitário de Saúde (+ a ordem de participação); ^††^AUXBUC = Auxiliar Bucal (+ a ordem de participação); ^‡‡^ENF = Enfermeiro (+ a ordem de participação); ^§§^APS = Atenção Primária à Saúde; ^||||^BAT-S = Sintomas secundários

Ainda na Figura 4, apresenta-se o fator 4 - Responsabilidade Social (item 14 com 0,742), gerado a partir de uma subdivisão do fator Benevolência (escala original). Na convergência, o código esforço expressa a preocupação ativa dos profissionais com os interesses do público e para com as pessoas usuárias da APS; a crítica às carências de condições físicas e estruturais que escapam da governabilidade e limitam a competência dos trabalhadores, expondo a necessidade da atuação das políticas públicas neste AT.

## Discussão

### A diferenciação dos achados conforme a ICE em português brasileiro

Observou-se que o instrumento utilizado para mensuração do CE nos ambientes da APS se molda conforme algumas características destes ambientes, portanto, não é absoluto. Os próprios tradutores da versão brasileira salientam que estudos futuros são necessários para identificar a adequação da estrutura proposta e seu poder preditivo do CE em diferentes organizações^(15)^.

Os profissionais da APS exprimem níveis coerentes (estáveis) de CE em seus AT. No entanto, partindo dos arquétipos de CE, onde ele é entendido como um atributo de uma organização que pode ser capturado por percepções individuais^(15)^, foi possível identificar os seguintes fatores dominantes nestes ambientes: Princípios e regras; Benevolência; Independência (individualismo/instrumentalismo) e Responsabilidade Social (desmembrado do fator Benevolência).

Um destaque do estudo foi a divisão do fator benevolência, gerando o novo fator de senso de comunidade/responsabilidade social. Isso representa uma contribuição à futuras pesquisas, pois, embora não expresse um conflito em relação ao autor original da escala, quando este situa o lócus cosmopolita da benevolência^(32)^, já em uma percepção do CE orientada para o sistema social e interesses coletivos. No entanto, ao ser observada uma relevância significativa do senso de comunidade/responsabilidade social, a ponto de sugerir uma discriminação maior e constitutiva de um novo fator, pode ser vislumbrada uma possível insuficiência de sua abordagem como simples lócus de benevolência, no grupo estudado.

Estudar o CE na APS requer mais análises tanto qualitativas quanto quantitativas do fenômeno, tendo em vista que o cenário é imerso em dimensões subjetivas e objetivas complexas e complementares. Entende-se que a forma como são atribuídos os significados e os entendimentos sobre a ética (sua presença e/ou ausência no cotidiano do trabalho) e na forma como a ética é “percebida e vivida”, agrega-se um sentido/valor ao ambiente de trabalho para cada profissional.

O fator 1 – “Princípios e regras” apresentou maior carga fatorial entre os profissionais. A afirmação que corresponde ao Item 11 do ICE – “Espera-se que as pessoas cumpram, acima de tudo, as leis e as normas profissionais”, a qual mostra, de forma geral, que os profissionais compreendem e esperam os cumprimentos das normas pertinentes de cada profissão, o que entendem como elementos constituintes do CE presentes nesses ambientes.

Esse achado também foi semelhante na atenção primária, entre enfermeiros e médicos que compartilhavam a percepção de um clima de equipe favorável quando há respeito aos objetivos da equipe; e o reconhecimento da enfermagem como categoria mais próxima aos ideais do processo de trabalho coletivo^(33)^.

A ética organizacional envolve coerência das ações com a missão, a visão e os valores da organização, o que torna necessária formas de autorregulação dinâmicas, valores compartilhados entre diversos atores e atenção ao seu cotidiano de interações sociais^(33)^. Os valores variam nos ambientes de saúde, inclusive pelas diferentes complexidades assistenciais ou tecnológicas, e diferem de regras ou normas constituintes da deontologia de cada profissão^(34)^. Valores e normas profissionais devem ser consideradas em suas distinções, mas, também, na forma como interagem e integram a formação de identidades profissionais^(35)^.

Tal apropriação e sentido local dado às normas sugere que intervenções éticas nos ambientes não precisam estar limitadas a regras e normas, mas sim estimuladas por meio da educação ética aos profissionais, de forma que eles possam exercer o julgamento de maneira honesta e responsável. A abertura ao diálogo entre equipes pode fazer com que o CE seja mais bem compreendido e modificado pelos profissionais.

No fator 2 – “Benevolência”, a afirmação “A nossa maior preocupação é o bem de todos os que trabalham nesta unidade de saúde” (item 2 do ICE) pode ter relação com o reconhecimento e satisfação com os gestores e com a adesão da equipe a objetivos comuns. A cooperação entre profissionais e o seu envolvimento com as pessoas usuárias que procuram a APS são elementos relatados como causas de satisfação no trabalho de gestores na estratégia saúde da família^(36)^. O achado expressa como a união dos profissionais e o senso de equipe reforçam o interesse comum e são ingredientes para manutenção de um ambiente favorável de trabalho.

A cooperação também é vista como elemento de subversão ao sofrimento imposto pelas condições adversas que há nos AT. O senso de coletividade pode ser equivalente à benevolência no sentido de contribuir para a construção de objetivos comuns, compreensão mútua e respeito a todos que atuam sob normas comuns^(37)^.

O fator 3 – “Independência e individualismo” expressou o senso de prioridade daquilo que é o interesse próprio dentro de determinada situação, ou o interesse na ação determinado pelo que o indivíduo acredita ser o correto. Foi possível detectar o grau de adesão dos dados qualitativos ao fator no item “Neste serviço, as pessoas guiam-se pela sua própria ética pessoal” (item 17 do ICE).

O individualismo pode se sobrepor aos interesses do público ou da equipe, contribuindo para a sobrecarga de trabalho e excesso de demanda para aos colegas de trabalho. O excesso de demandas próprias e de terceiros favorece a ocorrência de efeitos indesejados tanto sobre ambiente quanto sobre o público que é atendido, além de insatisfação por parte do profissional^(38)^. O mesmo ocorre no esgotamento das relações, onde episódios conflituosos e eticamente negativos podem provocar um clima desfavorável e até o distresse moral^(39)^.

Obviamente, a insatisfação, sobrecarga de trabalho, desgastes e sofrimento são fenômenos complexos, com múltiplas causas e consequências, cabendo aqui apenas a indicação da possibilidade de vinculação com processos de exaustão da cooperação e estímulo a respostas individualistas, de defesa de interesses pessoais ou de autopreservação em tais AT.

O problema de acesso a oportunidades de educação ética aos trabalhadores é uma realidade nestes ambientes. Para a categoria dos ACS, ainda não há um código de ética ou entidade com atribuição de fiscalização do exercício profissional, muito menos a percepção desse respaldo ético da categoria profissional diante de situações recorrentes nos atendimentos de pessoas usuárias da APS. Tal lacuna não impede que tais trabalhadores avaliem e formulem juízos morais sobre o ambiente de acolhimento e assistência^(40)^. Daí a urgência de investimentos e valorização da educação permanente em saúde (EPS) na prática concreta dos serviços de saúde, com a inclusão do ACS em espaços de aprendizados coletivos^(41)^.

Quando não há estímulos ou ações de EPS relacionados às situações que requerem competência ética, as ações/inações éticas passam despercebidas, omitidas, negligenciadas e, portanto, sem resolutividade. Esse aspecto pode trazer sofrimento moral a quem percebe tais lacunas, nesses casos, geralmente, o profissional enfermeiro sensibilizado a perceber problemas morais, pois sua formação perpassa tais definições e entendimentos^(42)^.

No Fator 4 – “Senso de comunidade/responsabilidade social”, aportou-se uma contribuição adicional do estudo misto para uma abordagem mutuamente complementar entre os fatores 2 e 4. O fator 4 é apresentado como novo componente, derivado do segundo, não por divergência, mas sim por aprofundamento, uma vez que uma interpretação possível é de que o senso de comunidade/equipe ou reponsabilidade social se mostra como uma forma de concretizar o bem comum (benevolência – fator 2). Ao mesmo tempo, uma forma de benevolência interna (entre pares) consolida e integra equipes de trabalho para que possam melhor exercer seu papel social (senso de comunidade se desdobrando da benevolência). A responsabilidade social conforma-se como um critério ético, não apenas como lócus de análise ou uma característica cosmopolita do CE^(15)^, conforme inicialmente proposta por Victor e Cullen^(32)^. Assim, sugere-se que o simples lócus cosmopolita pode ser insuficiente para explicar os elementos trazidos pelo estudo misto, podendo se constituir em um construto próprio.

A responsabilidade social é vista como um atributo do reconhecimento e pertencimento a um serviço e sistema público de saúde – de percepção de si como apto a orientar, assistir e ajudar pessoas que buscam atendimento. É uma noção apropriada por quem trabalha com comunidades, nutre expectativas de resolutividade e enfrenta precariedades (estruturais, materiais e de segurança) e impotência nesses profissionais, pois extrapolam os limites da sua unidade ou campo de atuação^(37)^.

Apesar da riqueza dos achados, o estudo teve como limitação a ausência da ampliação da estratificação amostral de algumas ocupações que permitissem explorar diferenças com os fatores envolvidos. Além disso, há que se reconhecer que o momento retratado estava altamente impactado pela pandemia de COVID-19.

Estudos futuros com intuito de confirmar a efetivação do ICE em demais ambientes da APS, noutras regiões do país, com aspectos diferenciados do que foi encontrado na cidade do norte amazônico, poderão dar novas perspectivas para tipologias do CE nos AT em saúde. Embora sem um objetivo de estudo metodológico, a aplicação do instrumento permite sugerir que ele sofra adaptações para o cenário da saúde, incluindo o fator responsabilidade social; e ajustes linguísticos (exemplo: pessoas usuárias ao invés de clientes).

O estudo contribui para uma compreensão mais aprofundada sobre o conceito de CE e seu potencial para subsidiar transformação nos AT, em especial na APS. A captação de múltiplos dados e visões é útil para subsidiar iniciativas de aprimoramento destes AT para seus trabalhadores e pessoas usuárias. A qualidade dos resultados do trabalho e oferta de cuidados não pode ser alcançada sem a devida atenção sobre os trabalhadores e os ambientes em que atuam, incluindo as dimensões éticas destes ambientes. A contribuição do estudo para a Enfermagem se evidencia por serem estes os trabalhadores grandemente afetados por seus AT e, também, pelo papel de protagonismo que assumem para que sejam transformados, desde que a interseção entre ética e AT sejam devidamente reconhecidas e problematizadas. Além disso, o estudo misto conseguiu projetar uma configuração interpretativa ampliada aos resultados obtidos com o inventário, apoiando a expressão da complexidade do objeto, de modo a fornecer resultados indicativos de uma futura adaptação do instrumento para o campo da saúde.

## Conclusão

Vários fatores relacionados à aferição do CE mostraram-se pertinentes nos ambientes da APS analisados: a relevância dos princípios e regras, quando é referida aderência às normas pertinentes aos códigos deontológicos de cada profissão; à benevolência, ao referir o bem-estar dos sujeitos que ali trabalham como orientador de ações e escolhas; a independência e instrumentalismo, que traz a discussão sobre os agentes morais e os conflitos que podem surgir no AT quando a atitude ética é negligenciada ou desconhecida e; por fim, o senso de comunidade/responsabilidade social, derivada da benevolência como preocupação de prestar um bom serviço público e favorecer o trabalho coletivo integrado.

A integração dos dados quantitativos e qualitativos tendo como ponto de fusão os quatro construtos/fatores ampliou e aprofundou o entendimento sobre o objeto em sínteses analíticas que: – reforçam a importância da ética profissional/profissionalismo, ancorada tanto em normas profissionais quanto institucionais; – a força da benevolência que se dirige tanto à comunidade como às relações entre profissionais na equipe; – o exercício de julgamentos morais impondo desafios, mas também a imagem de agência moral fundamental para que se percebam como sujeitos morais das ações; por fim, uma importante distinção dada ao senso de comunidade (entre profissionais) e responsabilidade social, como constituinte de um CE no cenário estudado. Tal distinção do quarto construto foi uma contribuição importante e sustentada por dados quantitativos e qualitativos, o que é desejável em aplicações de instrumentos validados, os quais requerem subsídios para o seu aprimoramento.

O estudo trouxe à discussão um cenário pouco explorado em termos de CE, a APS e um município da região amazônica. O objeto de estudo deve, necessariamente, considerar as particularidades de cada ambiente – só assim terá um potencial transformador de cenários, qualificando as experiencias de trabalho e seus resultados para pessoas e comunidades usuárias. As dimensões éticas de ambientes e relações na APS têm no CE uma ferramenta conceitual e analítica relevante, enquanto estudos mistos apoiam a compreensão da sua complexidade.

## Data Availability

O conjunto de dados deste artigo está disponível nos seguintes links: https://www.dropbox.com/scl/fi/ghsc4rw2f73k8bayct5om/Relat-rio_21072021_FATOR1.pdf?rlkey=q8iw7p7uqsuewvuneddefc721&st=yw2jjg7c&dl=0 https://www.dropbox.com/scl/fi/szyv293q7vss8xsf3oywi/Relat-rio_21072021_FATOR2.pdf?rlkey=9anfauf9xnr2ml54pcvjaz0r8&st=fasfir3h&dl=0 https://www.dropbox.com/scl/fi/kb5wrpm1ar7ycapkjk03p/Relat-rio_21072021_FATOR3.pdf?rlkey=pjrxfroumomx47ls5gbhlj7go&st=ppk2ee2r&dl=0 https://www.dropbox.com/scl/fi/ffxf9ybpp442gwea406o0/Relat-rio_21072021_FATOR4.pdf?rlkey=g5t0pa17v91wa81c4brl6aicl&st=x3vr3d00&dl=0 https://drive.google.com/file/d/1mApBFHq7PF6qADF9mzkAhq2KDyuCQJwN/view?usp=drive_link

# Translation: Clima ético en el entorno de trabajo en la atención primaria de la salud: un estudio mixto*

**DOI:** 10.1590/1518-8345.7499.4755

**Authors:** Lenna Eloisa Madureira Pereira, Laura Cavalcanti de Farias Brehmer, José Luís Guedes dos Santos, Graziele de Lima Dalmolin, Lucas Lorran Costa de Andrade, Flávia Regina Souza Ramos

**Affiliations:** 1 Universidade Federal do Pará, Departamento de Enfermagem, Belém, PA, Brasil. Universidade Federal do Pará Departamento de Enfermagem PA Belém Brasil; 2 Becaria (o) del Conselho Nacional de Desenvolvimento Científico e Tecnológico (CNPq), Brasil. Becaria (o) del Conselho Nacional de Desenvolvimento Científico e Tecnológico Brasil; 3 Universidade Federal da Santa Catarina, Departamento de Enfermagem, Florianópolis, SC, Brasil. Universidade Federal da Santa Catarina Departamento de Enfermagem SC Florianópolis Brasil; 4 Universidade Federal de Santa Maria, Departamento de Enfermagem, Santa Maria, RS, Brasil. Universidade Federal de Santa Maria Departamento de Enfermagem RS Santa Maria Brasil

**Keywords:** Atención Primaria de Salud, Ética, Trabajo, Encuestas y Cuestionarios, Enfermería, Servicios de Salud

## Abstract

analizar el clima ético en el entorno de trabajo de la atención primaria de la salud en un municipio de la región amazónica, mediante la articulación de datos cuantitativos y cualitativos.

estudio mixto convergente paralelo. Los datos cualitativos de entrevistas con 46 profesionales se sometieron a análisis temático reflexivo. Por su parte, los datos cuantitativos se obtuvieron con 170 profesionales por medio del Inventario de Clima Ético. El análisis factorial exploratorio probó el modelo del Inventario de Clima Ético, proponiendo una nueva configuración de los ítems, con poder explicativo superior al mantenido en el análisis factorial confirmatorio. La integración de los datos fue independiente y se presentó en *joint display*.

los ítems con mayor carga factorial de los cuatro factores del Inventario de Clima Ético (1. principios y reglas, 2. benevolencia, 3. independencia/individualismo, y 4. sentido de comunidad/responsabilidad social) se analizaron en fusión con la categoría cualitativa, con múltiples expresiones de la subjetividad y de la ética en el entorno de trabajo, no observándose divergencias entre los datos, sino solo convergencias y complementación en síntesis analíticas por factor.

la integración de los datos amplió la comprensión del clima ético en un escenario poco explorado, demostrando la distinción de esa realidad en términos de los constructos analizados y la fuerza de los elementos éticos como herramienta conceptual y analítica de los entornos de trabajo.

## Destacados

**(1)** Los profesionales de la Atención Primaria de la Salud presentan un clima ético cohesionado en sus entornos de trabajo. **(2)** Los valores varían en los entornos de salud, debido a la diversidad asistencial y tecnológica. **(3)** La cooperación subvierte el sufrimiento impuesto en entornos de trabajo adversos.

## Introducción

El clima ético (CE) fue definido en la década de 1980, por Victor y Cullen, como un conjunto de comportamientos, emociones y percepciones que caracterizan a una organización y son moldeados por elementos diversos, como valores profesionales, normas, concepciones y tradiciones. Fue redefinido en Enfermería, por Olson, como las percepciones del equipo de enfermería sobre cómo se abordan o resuelven las cuestiones éticas en su entorno de trabajo (ET) específico, en este caso, el hospitalario^(1)^. Tales percepciones problematizan las condiciones organizacionales para ayudar a los trabajadores en las reflexiones éticas cotidianas, que involucran la asistencia, la gestión, las políticas y las decisiones sobre problemas éticos; de ahí que el CE promueva la satisfacción profesional y un entorno saludable y seguro para el cuidado, mientras que el CE negativo es un predictor de riesgos de agravamiento de la salud del trabajador^(2)^.

A lo largo de los años, los estudios sobre CE se han perfeccionado tanto en el área de la administración y la psicología como en el área de la salud, aunque en este campo se han concentrado en escenarios hospitalarios^(3)^ y en relaciones con otros factores, como salud del trabajador^(2)^, habilidad de toma de decisiones y capacitación en comunicación interprofesional^(4)^; compromiso de enfermeras(os)^(5)^; distrés moral^(6-7)^ y fatiga por compasión^(7)^; intención de abandonar el trabajo^(8)^; satisfacción laboral^(1)^, entre otros^(3,9)^.

En el campo de la Enfermería y del ámbito hospitalario se destaca el estudio pionero de la escala *Hospital Ethical Climate Survey* (HECS)^(9)^, de autoría de la enfermera Linda Olson, la más utilizada según una revisión que analizó 61 estudios sobre CE en 21 países^(3)^. Esta misma revisión evidenció la brecha de análisis en escenarios de la Atención Primaria en Salud (APS), al observar variabilidad únicamente en unidades hospitalarias, aun cuando se utilizaron escalas generales, como el *Ethical Climate Questionnaire* o ECIndex (ECQ/ECI); además del mayor número de estudios con muestras de enfermeras(os)^(3)^. El ECQ posee versiones traducidas para diferentes países^(3,10-11)^; el HECS también cuenta con traducciones y aplicaciones en los cinco continentes, incluido el portugués brasileño^(3,9,12)^, esta última con la colaboración de la autora de la versión original. Cabe mencionar además la *Social Capital and Ethical Climate at the Workplace Scale* (SEW)^(13)^, que analiza las intenciones de rotación, lesiones y accidentes de trabajo entre cuidadores profesionales; y el *Ethical Decision-making Climate Questionnaire* (EDMCQ)^(8)^, que evalúa el CE en unidades de cuidados intensivos.

El análisis del CE cuenta con diferentes enfoques y marcos teóricos, de acuerdo con los factores asociados al concepto. La tipología del CE de Victor y Cullen se basó en una matriz 3×3 que combina tres criterios éticos (principio, benevolencia y egoísmo) con tres locus de análisis (individual, local y cosmopolita)^(14)^, produciendo nueve arquetipos. Según este referencial, en términos de criterios éticos, un CE contempla la benevolencia cuando se fundamenta en la “preocupación por los otros/bienestar del otro”; los principios y reglas, cuando está afectado por la adhesión del individuo a las normativas y reglamentos de su profesión y/o determinados por la organización; y se configura por la independencia/individualismo (denominada inicialmente “egoísmo”), cuando la actuación se orienta por creencias e intereses personales^(11,14)^. En términos de locus de análisis, el individual se refiere a los intereses personales; el local, cuando recae sobre los intereses del sistema interno de la organización/grupos; y el cosmopolita, cuando involucra el sistema social externo a la organización. La representación gráfica de los nueve arquetipos, según la propuesta original de Victor y Cullen, consta en la Figura 1, siendo que el término “egoísmo” dio origen al constructo independencia/instrumentalismo y el término “principios” originó el constructo principios y reglas.

**Figura 1-:** Locus de análisis ético en las decisiones: individual, local y cosmopolita. Belém, PA, Brasil, 2025

		**Locus de análisis**		
		INDIVIDUAL	LOCAL	COSMOPOLITA
**Criterio ético**	EGOÍSMO	Interés propio	Beneficios/intereses de la empresa	Eficiencia
	BENEVOLENCIA	Amistad	Interés del equipo	Responsabilidad social
	PRINCIPIO	Interés propio	Reglas y procedimientos de la organización	Códigos profesionales y leyes

Fuente: Ribeiro, et al, 2016^(15)^

A partir de ese enfoque, Victor y Cullen construyeron un instrumento de medición, el Inventario de Clima Ético (ICE, en inglés ECQ/ECI), con adaptación y validación en versión brasileña^(15)^ en el ámbito de la psicología y la gestión de personas. Para el campo de la salud y la Enfermería existen adaptaciones de la versión original en lenguas extranjeras, pero no para Brasil^(1)^.

Los ET en salud se diferencian según contextos culturales, complejidad y tipos de servicios, equipos actuantes, entre otros. En el escenario hospitalario, existe una porción considerable de estudios pertinentes al CE^(3,9,16-18)^. Por otro lado, no existen instrumentos específicamente elaborados para evaluar el CE en la APS. A partir de esa laguna identificada en la literatura, se justifica la importancia de relacionar las experiencias de los trabajadores en ET en la APS con la evaluación del CE realizada por trabajadores de esos escenarios.

En el escenario brasileño, se destaca el hecho de que el país cuenta con un sistema de salud público y universal que, a pesar de los avances, aún enfrenta desafíos relacionados con disparidades socioeconómicas, escasez de recursos, integración de servicios y diversas barreras de acceso a la atención médica^(19)^. Además, Brasil cuenta con vastas áreas geográficas remotas, como en la Amazonía, con oferta de servicios limitada, falta de profesionales de la salud y de infraestructura adecuada. La escasez de recursos en esas áreas puede traducirse en largas distancias que recorrer para obtener atención y en la falta de equipos y medicamentos esenciales^(19)^.

La evaluación del CE tiene el potencial de revelar cómo condiciones subjetivas y éticas específicas de cada contexto son percibidas por los trabajadores en sus impactos sobre sí mismos, sobre el trabajo y sobre el cuidado. Ante ello, es fundamental dirigir este tipo de investigación hacia campos aún poco explorados en el sistema de salud, como los servicios de APS y aquellos situados fuera de los grandes centros urbanos de Brasil. Debido a la complejidad que envuelve el fenómeno en cuestión, un enfoque monométodo se muestra limitado para captar sus múltiples dimensiones. En este sentido, el uso de un diseño de investigación de métodos mixtos se considera más adecuado, pues posibilita integrar diferentes perspectivas analíticas, ampliando la profundidad y el alcance de la comprensión del objeto investigado.

Así, se delinearon, en este estudio, tres preguntas de investigación articuladas al diseño de métodos mixtos: la pregunta cuantitativa buscó identificar qué factores o criterios evaluados por el ICE presentan relevancia en un escenario de la APS; la pregunta cualitativa investigó cómo se expresa la dimensión ética de los ET de la APS, según la perspectiva de los trabajadores; y la pregunta integradora procuró comprender las convergencias y/o divergencias entre los resultados obtenidos por medio del ICE y los relatos de los trabajadores sobre el ET en la APS de un municipio de la región amazónica.

Ante este panorama, el objetivo de este estudio fue analizar el clima ético en el entorno de trabajo de la atención primaria de la salud en un municipio de la región amazónica, mediante la articulación de datos cuantitativos y cualitativos. Es decir, explorar convergencias y divergencias entre la evaluación realizada por medio del ICE y los relatos sobre el ET de los trabajadores de la APS.

## Método

### Diseño del estudio

Se trata de un estudio mixto convergente paralelo (CUANTI-CUALI), en el que los datos cualitativos y cuantitativos se recopilan concomitantemente y con la misma atribución de peso. Se utilizó el enfoque de métodos mixtos por permitir la integración de datos cuantitativos y cualitativos, atendiendo a la necesidad de comprender el fenómeno del CE, foco de este estudio, de forma más amplia y profunda^(20-21)^. El *Mixed Methods Appraisal Tool* (MMAT), versión 2018^(22)^, se utilizó como guía metodológica^(23)^.

### Lugar y población del estudio

El escenario del estudio fue la APS de un municipio perteneciente a la región metropolitana de Belém, capital del estado de Pará, Brasil. Para atender a más de 500.000 habitantes, el municipio estudiado dispone de 12 unidades básicas de salud, distribuidas en cinco regiones. Se seleccionaron dos regiones que reúnen siete unidades municipales de salud, por atender a los mayores contingentes poblacionales. La población del estudio comprendió el número total de trabajadores de las siete unidades, totalizando 444 trabajadores.

### Estudio cuantitativo: muestra, instrumento, recolección y análisis de los datos

La fase cuantitativa fue un estudio transversal analítico que abarcó a toda la población (trabajadores de las siete unidades). Los criterios de inclusión fueron: servidores concursados y/o contratados, con tiempo de actuación de al menos dos meses. Se excluyeron servidores en vacaciones o licencia, vigilantes y residentes de las unidades investigadas. Mediante cálculo muestral para población finita, considerando la población de 444 trabajadores con error muestral de 5% y nivel de confianza de 95%, se estimaron 169 participantes.

Se empleó un muestreo por conveniencia, invitándolos a participar a todos los trabajadores que estaban presencialmente en las siete unidades en los días de recolección de datos y, también, a aquellos a quienes se tuvo acceso vía *WhatsApp*^®^. Fueron abordados 214 trabajadores, de los cuales 44 declinaron, constituyéndose la muestra por 170 participantes, de las siguientes profesiones/cargos en las siete unidades: enfermeros, técnicos y auxiliares de enfermería, médicos, demás profesionales de nivel superior (trabajador social, psicólogo, terapeuta ocupacional, nutricionista, odontólogo), agentes comunitarios de salud (ACS), demás profesionales de nivel medio (técnico y auxiliar bucal, técnico en patología clínica, técnico en radiología), gestores y asistentes administrativos, agentes de servicios generales. Los términos trabajadores o servidores engloban a la mayoría de los participantes con profesiones reconocidas y a quienes ejercen una ocupación o cargo independientemente de la formación, como los tres últimos citados.

El instrumento utilizado para la recolección de datos fue el ICE (*Ethical Climate Index*) traducido, adaptado y validado para la realidad brasileña^(15)^. La versión original, con 36 ítems, ya había pasado por revisiones, incluso en portugués, de modo que el instrumento adoptado^(15)^ fue validado en tres constructos/factores y 19 ítems: benevolencia (9 ítems), principios y reglas (6 ítems) e independencia e instrumentalismo (4 ítems), manteniendo coherencia con los arquetipos iniciales (Figura 1). Las pruebas estadísticas para la extracción de los factores fueron Kaiser y método de ejes principales (PAF) con rotación Promax. Cada ítem se mide por escala Likert de 6 puntos; del 1 (completamente falsa) al 6 (completamente verdadera). El instrumento no sufrió cambio de contenido, tan solo el ajuste de un término en sus preguntas: el término “empresa” fue sustituido por “unidades de salud”. Dado que el instrumento original contempla organizaciones en general y, en esta aplicación específica, el escenario se refería únicamente a la APS, tal modificación se realizó para una mejor comprensión de los participantes, de modo que las demás afirmaciones permanecieron inalteradas. Así, se optó por no realizar una nueva adaptación y validación, incluso considerando que su uso podría indicar tal necesidad futura. Se obtuvo la autorización de uso con la primera autora de la versión brasileña del instrumento^(15)^.

La recolección de datos ocurrió tras el reclutamiento mediante divulgación presencial en las unidades y vía *WhatsApp*^®^ a contactos obtenidos por indicación de gestores y trabajadores. El diligenciamiento del instrumento, tras la firma del CI, fue en línea, individual, en el período de agosto de 2020 a marzo de 2021, vía formulario de *Google Forms*, conteniendo cuestionario sociolaboral (edad, género, estado civil, hijos, situación conyugal, posgrado y tipo y número de vínculos) y el ICE^(14)^.

El análisis estadístico se realizó con el *software Statistical Package for the Social Sciences* (SPSS) versión 25 e IBM *Amos Graphics* v.20^(24)^. Inicialmente, se efectuó un análisis de verificación de la aplicabilidad de la escala al escenario estudiado, con el empleo de ecuaciones estructurales e índices de análisis factorial confirmatorio (AFC) y, posteriormente, análisis factorial exploratorio (AFE). La descripción de los resultados acerca de la evaluación del CE en esta población fue explorada detalladamente en una publicación previa^(25)^, incluyendo pruebas de comparación entre análisis de correlación, cuando se trató de relacionar factores del ICE y variables categóricas o cuantitativas.

En este estudio, se utilizó el modelo de ecuaciones estructurales (*Structural Equations Modeling* – SEM) para poner a prueba el modelo del ICE en el contexto de la APS considerando la estructura y contenido del instrumento. Los índices de ajuste y parámetros para evaluar el modelo, que propusieron una nueva configuración para los ítems, con poder explicativo superior al mantenido en la AFC, fueron: razón chi-cuadrado por grados de libertad (χ²/gl) menores que 3; (A)GFI representando el (*Adjusted*) *Goodness of Fit* mayores o iguales a 0,95; RMSEA menores que 0,06, donde el valor del intervalo de confianza del 90% de ese indicador no debe superar 0,10; valores de *Normed Fit Index* (NFI), *Comparative Fit Index* (CFI) y *Tucker-Lewis Index* (TLI) mayores que 0,9^(26-27)^.

Se empleó también, para adecuar el modelo al escenario y a la población, la verificación del AFE. Se utilizaron las pruebas de Kaiser-Meyer-Olkin (KMO) para verificar el criterio de adecuación muestral (valores iguales o menores de 0,5 se consideran inadecuados para el análisis factorial) y la prueba de esfericidad de Bartlett (p<0,05) para la evaluación de la matriz de correlación. Los valores de comunalidad y carga factorial aceptables deben situarse por encima de 0,400; sin embargo, para la integración de los datos en el estudio mixto, se consideraron las preguntas que presentaron cargas superiores a 0,700^(28)^.

### Estudio cualitativo: muestra, instrumento, recolección y análisis de los datos

La fase cualitativa tuvo carácter exploratorio-descriptivo y se realizó con entrevistas a 46 trabajadores de la APS. Se adoptó un muestreo por conveniencia, siguiendo los mismos criterios de inclusión y exclusión de la etapa cualitativa.

El instrumento fue un guion de entrevista compuesto por siete preguntas abiertas, que fue sometido a ajustes por tres investigadoras y probado con dos trabajadores de los mismos servicios, uno con formación de nivel medio y otro de nivel superior, que no integraron la muestra. El guion de entrevista contenía siete preguntas abiertas sobre: percepción y elementos de un entorno saludable/positivo; percepción sobre entorno ético; identificación de condiciones de un entorno saludable/positivo y de un entorno ético en su escenario de trabajo; cómo contribuir a entornos éticos; qué situaciones son limitantes y qué puede mejorarse para tornar los entornos éticos.

Tras la firma del CI, las entrevistas individuales fueron realizadas presencialmente, por la primera autora, entre agosto y diciembre de 2020, conforme a la disponibilidad de los participantes y tuvieron una duración media de quince minutos. El registro de los datos se realizó mediante la aplicación digital Grabadora de Voz^®^ Versión 3 (42.0). Los audios fueron íntegramente transcritos por la entrevistadora y organizados en el *software* Atlas.ti, cuyas funcionalidades apoyaron el análisis cualitativo en las seis fases del análisis temático reflexivo (ATR) (familiarización de los datos; generación de códigos iniciales; búsqueda de temas; revisión de temas; definición de temas; elaboración del informe)^(29-30)^. Todo el proceso fue sometido a doble verificación hasta la categorización final (primera y última autora). Del libro de códigos (*codebook*), la categoría “Elementos subjetivos y éticos”, con magnitud (Mt) de 163 extractos, fue posteriormente integrada a los datos cuantitativos de los 19 ítems del ICE.

Previamente a la integración, todos los datos cualitativos fueron codificados y analizados de forma independiente a la finalidad de integrar. De este análisis derivaron dos grandes redes temáticas que agrupan categorías o agrupamientos de códigos, refiriéndose a: 1) Entorno de trabajo (ET) y salud, o formas de ver la realidad de su entorno y proyectar ET deseables; esta red reunió 322 extractos/citas (magnitud) y tres categorías (condiciones materiales, de gestión y de comunicación, con 10 códigos en total); 2) Componentes subjetivos y relacionales del AT en la APS, compuesta por tres categorías con magnitud total de 311 extractos y 30 códigos, que tratan de las múltiples expresiones de la ética y la subjetividad (concepciones y cuestionamientos éticos, problemas éticos y atributos para el CE), de las relaciones de trabajo y de los equipos profesionales y de las percepciones y experiencias de sí en el trabajo (como apoyo, pertenencia, esperanza, pero también irrespeto y desvalorización).

### Estudio mixto: procedimientos de fusión de los datos

Tras el análisis individual e independiente de los datos cualitativos y cuantitativos, se procedió a la etapa de integración, que consiste en producir un resultado síncrono y consistente mayor que la suma de las partes cualitativa y cuantitativa^(31)^.

Algunos procedimientos estadísticos fueron especialmente útiles para definir resultados aplicables a la integración o fusión de datos CUANTI-CUALI, en diferencias y semejanzas (diseño convergente)^(23)^. Como ya se mencionó, a partir de la verificación del modelo del ICE en el escenario de la APS, a partir del AFE, se consideraron solo las preguntas que presentaron mayor impacto en sus respectivos factores, evaluadas por la mayor carga factorial (>0,700).

Para el presente recorte mixto, se asociaron las preguntas de mayor carga factorial de los cuatro factores del ICE con los 19 códigos (subcategorías) cualitativos estudiados. Un nuevo análisis recayó sobre 163 extractos, inicialmente contenidos en la categoría múltiples expresiones de la ética y subjetividad, ahora revisados por sus contribuciones al profundizamiento de datos cuantitativos relevantes. Es decir, del conjunto de datos cualitativos, solo se produjo un agrupamiento para esta finalidad de integración, preservando la originalidad de los datos cualitativos de otros agrupamientos, susceptibles de ser explorados en publicaciones exclusivamente cualitativas.

Los datos cualitativos y cuantitativos fueron integrados vía *joint display*. Cada asociación de ideas, convergentes y/o divergentes, según los testimonios asociados al ICE, fue insertada en dominios. Se constituyó, así, un enfoque de entrelazamiento a nivel del método mixto, es decir, hilos de encuentros con base tema por tema, o concepto por concepto^(21)^.

### Aspectos éticos

El proyecto de investigación recibió anuencia institucional de la Secretaría de Salud del municipio y obtuvo aprobación del Comité de Ética en Investigación de la *Universidade Federal de Santa Catarina*, bajo dictamen n.º 4079091, de 09/06/2020, CAAE 32493920.4.0000.0121, en consonancia con la Resolución del Consejo Nacional de Salud n.º 466/2012.

## Resultados

Los resultados de la etapa cuantitativa fueron reportados en una publicación anterior^(25)^, en la cual los datos e interpretaciones, característicos de un estudio transversal y correlacional, no son objeto del presente artículo. Por ejemplo, los datos de correlación son exclusivos de aquel recorte, así como las medias de intensidades por cada ítem, manteniendo la configuración original de la escala en tres factores. En el presente recorte (mixto) se presentó una nueva configuración, en cuatro factores, obtenida en pruebas complementarias y como contribución exclusiva y respaldada por la integración CUANTI-CUALI.

De hecho, el ya referido entrelazamiento, que explora hilos de encuentros entre los enfoques en torno a un tema/concepto, supera su mera sumatoria y, por ello, se recomienda el informe en artículos distintos, no tratándose ya de abordar la completitud de los resultados CUANTI-CUALI^(19)^. Una de las ventajas de los estudios de métodos mixtos es la posibilidad de desdoblar diferentes productos académicos a partir de un único proyecto. En general, pueden generarse hasta cuatro publicaciones distintas: una con los resultados cuantitativos, otra con los resultados cualitativos, una tercera con el análisis integrado de los datos y, por último, un relato metodológico que destaque las innovaciones en la concepción y conducción del estudio. Es importante destacar que esta estrategia no se configura como repetición de contenido, sino como una utilización legítima y planificada del banco de datos, en la que cada publicación posee identidad propia, con recortes analíticos y objetivos específicos, respetando los principios éticos y científicos de la investigación^(19)^.

Los participantes de la etapa cuantitativa fueron 170 trabajadores de la salud, con edad media de 39 años (DE± 10,2), mayoritariamente mujeres (78,1%), con compañero (DE± 49,4) e hijos (65,7%), con nivel de formación superior (57,0%), concursados en régimen de 40 horas semanales (62,0%); sin otros vínculos laborales (64,1%).

Los parámetros de la AFC, considerando el modelo original del ICE para el escenario estudiado, presentaron los siguientes valores: chi-cuadrado (χ²) de 293,1 con 146 grados de libertad (df), resultando en una razón χ²/df de 2,00. El *Goodness of Fit Index* (GFI) fue de 0,854, y el *Adjusted Goodness of Fit Index* (AGFI) alcanzó 0,810. El *Normed Fit Index* (NFI) registró 0,817, mientras que el *Tucker-Lewis Index* (TLI) obtuvo 0,880. El *Comparative Fit Index* (CFI) fue de 0,897, y el *Root Mean Square Error of Approximation* (RMSEA) presentó un valor de 0,075, con intervalo de confianza del 90% entre 0,063 y 0,087.

La razón entre la estadística chi-cuadrado y los grados de libertad presentó valores dentro de lo esperado. Los parámetros de la AFC se mostraron muy próximos a lo esperado, resultado que sugirió probar un nuevo modelo mediante AFE en el presente estudio.

En el AFE, los indicadores confirmaron la adecuación del ICE en cuatro dimensiones (Tabla 1), con valor de KMO (por encima de 0,8) que sugiere adecuación muestral aceptable para el AFE, así como prueba de esfericidad de Bartlett significativa (p<0,01) indicando la factorabilidad de la matriz de correlación. Los valores de carga factorial variaron de 0,442 a 0,834, indicando que una proporción moderada a sustancial de la varianza en cada ítem fue compartida con su respectivo factor.

El AFE, según la Tabla 1, presenta la nueva configuración de los ítems: factor principios y reglas (Facto 1 – PR), que mantiene seis ítems (preguntas 8, 10, 11, 12, 13, 15); factor benevolencia (Factor 2 – NNV), con cinco ítems (1, 2, 3, 4, 6); factor independencia/individualismo (Factor 3 – IDP), con cuatro ítems (16, 17, 18, 19); y la constitución de un nuevo factor, denominado sentido de comunidad social (Factor – 4 RS), con cuatro ítems que antes pertenecían al factor benevolencia (5, 7, 9, 14). La Tabla 1 presenta los 19 ítems de la escala original, distribuidos en los cuatro factores, destacando aquellos que alcanzaron preponderancia (cargas factoriales superiores a 0,700), los cuales fueron utilizados para la integración con los datos cualitativos.

**Tabla 1-:** Análisis factorial exploratorio de los cuatro factores del Inventario de Clima Ético: principios y reglas (PR*), benevolencia (BNV^†^), independencia/individualismo (IDP^‡^) y sentido de comunidad/responsabilidad social (RS^§^). Belém, PA, Brasil, 2021

Í **tems**	**Componentes**			
	**Factor 1** PR*	**Factor 2** **BNV** ^†^	**Factor 3** **IDP** ^‡^	**Factor 4** **RS** ^§^
11.Se espera que las personas cumplan, por encima de todo, las leyes y las normas profesionales.	**0,815**	0,314	-0,141	0,105
12.En este servicio, se espera que las personas sigan estrictamente las normas legales y profesionales.	**0,772**	0,265	-0,067	0,229
10.Se espera la adhesión de todas las personas a las normas y a los procedimientos organizacionales.	**0,737**	0,273	-0,124	0,193
15.Es muy importante seguir estrictamente las reglas y procedimientos de la unidad de salud.	**0,736**	0,143	-0,086	0,048
13.Se espera que las personas hagan siempre lo que es mejor para los clientes y el público.	0,573	0,216	-0,276	0,343
8.Se espera que cada persona sea bien tratada cuando se toman decisiones.	0,512	0,331	-0,099	0,484
2.Nuestra mayor preocupación es el bien de todos los que trabajan en esta unidad de salud.	0,277	**0,834**	-0,013	0,173
1.La preocupación más importante del servicio es el bienestar de todas las personas que trabajan en él.	0,213	**0,753**	0,143	0,091
6.En la unidad de salud, nuestra mayor preocupación se concentra en lo que es mejor para la otra persona.	0,343	0,663	-0,308	0,198
3.En la unidad de salud, las personas se preocupan por el bien de cada uno de los demás.	0,367	0,488	-0,251	0,269
4.Las personas se preocupan mucho por lo que es globalmente mejor para los trabajadores.	0,281	0,442	0,057	0,440
17.En este servicio, las personas se guían por su propia ética personal.	0,087	-0,220	**0,783**	0,006
16.En este servicio, cada persona, por sí misma, decide lo que considera correcto e incorrecto.	-0,254	0,178	**0,717**	-0,066
18.En este servicio, las personas protegen, por encima de todo, sus propios intereses.	-0,153	-0,126	**0,716**	-0,354
19.Lo que importa en este servicio es lo que cada persona considera correcto o incorrecto.	-0,421	0,082	0,682	-0,125
14.Las personas se preocupan activamente por los intereses de los clientes y del público.	0,115	0,094	-0,257	**0,742**
9.Las personas de esta unidad de salud tienen un fuerte sentido de responsabilidad ante la comunidad exterior/externa.	0,229	0,352	-0,175	0,616
5.La primera preocupación en esta unidad de salud es lo que es bueno para cada individuo.	0,083	-0,018	0,475	0,590
7.Las personas de este servicio dan mucha importancia al espíritu de equipo.	0,239	0,356	-0,384	0,560

*PR = Principios y reglas; ^†^BNV = Benevolencia; ^‡^IDP = Independencia/individualismo; ^§^RS = Sentido de comunidad/responsabilidad social

En la etapa cualitativa, fueron entrevistados 46 profesionales, con características distribuidas de forma semejante a la muestra cuantitativa, quienes también fueron invitados a participar en la etapa cuantitativa o a indicar nuevos participantes.

El punto de fusión CUANTI-CUALI fueron las convergencias con los 19 ítems del ICE, que generaron un nuevo proceso analítico. Los códigos cualitativos se agruparon en: subjetividad o “sbj”; y ética o “etc”. Estos se describen a continuación, en la columna 2 de las Figuras 3 y 4.

Las múltiples expresiones de la subjetividad (sbj) se refirieron a las percepciones o efectos negativos que el ET producía en los trabajadores o que estos consideraban limitantes de un CE, por ejemplo, expresiones de miedo, desvalorización e irrespeto, negativismo en las formas de vislumbrar cambios y percibirse desatendido en sus necesidades. Al mismo tiempo, y paradójicamente, percepciones y efectos positivos eran atribuidos al esfuerzo y la proactividad individual y de equipo, al apoyo recibido, a la empatía, al reconocimiento y a la satisfacción a pesar de las dificultades; todos como elementos de un ET saludable y de un CE positivo.

A su vez, las expresiones de la ética (etc) se mostraron como la base o las justificaciones de las posturas y acciones profesionales, ya basadas en la ética personal (valores, principios y formación familiar), pero, principalmente, en la ética profesional (principios y normas, de carácter deontológico, derechos y deberes). Además, se reportaron dudas morales (inseguridad y falta de sustento), conflictos éticos (situaciones de desacuerdo moral entre profesionales o con usuarios) que, aunque dificultaban, no impedían manifestaciones de agencia moral, o condiciones de actuar conforme a un juicio moral y en dirección a la promoción del CE.

La Figura 2 presenta el análisis de relaciones (coocurrencia) de cada uno de los cuatro factores del ICE con los códigos cualitativos de la red temática “ética y subjetividad”, obtenidos con el auxilio del *software* Atlas.ti^®^.

**Figura 2-:** Análisis de coocurrencia entre códigos cualitativos e ítems de los 4 factores del Inventario de Clima Ético. Belém, PA, Brasil, 2021

Los resultados integrados se presentan en dos figuras que destacan, para cada factor de la escala, síntesis relevantes del análisis cuantitativo y convergencias y divergencias observadas cuando se articulan con subcategorías de la fase cualitativa, abordando específicamente expresiones e importancia de la ética profesional/personal y percepciones y experiencias en el ET que involucran el CE.

Solo los ítems con mayores valores de carga factorial (≥ 0,700) se describen en columnas, según el AFE (Tabla 1), seguido por la identificación de la categoría temática cualitativa con expresiva concentración de ítems (columna 2) y ejemplos de extractos discursivos que ejemplifican tal interpretación convergente (+) o divergente (-) en la columna 3. Como se observa en la Figura 2, hay coocurrencia con prácticamente todas las categorías, pero la integración (Figura 3) explora la categoría con mayor concentración de relaciones.

El factor “principios y reglas” (ítems 11 con 0,815; 12 con 0,772; 10 con 0,737 y 15 con 0,736) mostró expresivas convergencias con la categoría “ética profesional” (mayor concentración), convergiendo con los discursos sobre la importancia de los códigos, normativas y regulaciones profesionales e institucionales para un CE positivo.

En el factor benevolencia (ítems 2 con 0,834; 1 con 0,753) se observa convergencia con la percepción del entorno de trabajo saludable que destaca la preocupación por el bien de todos los que trabajan en la unidad de salud o que allí son atendidos.

**Figura 3-:** Integración de resultados entre Factor (1) Principios y reglas (CUANTI^‡^) y categoría Ética profesional (CUALI^§^), y Factor (2) Benevolencia (BNV^‡‡^) (CUANTI^‡^) y categoría Percepciones y experiencias en el entorno de trabajo (CUALI^§^). Belém, PA, Brasil, 2021

Factor 1 – Principios y reglas (PR*)		
**ICE** ^†^ **– CUANTI** ^‡^	**Categor** í **a CUALI** ^§^	**Convergencias (+) o Divergencia (-)**
Se evidencia una importante valoración de: Leyes y normas profesionales (expectativa de cumplimiento y seguimiento estricto) (ítems 11 y 12); Adhesión de todas las personas a las normas y a los procedimientos organizacionales (ítem 10); Reglas y procedimientos de la unidad de salud (seguimiento estricto) (ítem 15).	Coocurrencia concentrada de los ítems 11, 12 y 10 con las categorías “Ética profesional”: valores, principios y normas que rigen el ejercicio profesional y el trabajo en equipos multiprofesionales, pero que pueden generar conflicto ético cuando las obligaciones se incumplen.	**(+):** *Es mantener el sigilo de las cosas, de lo que ocurre aquí. A veces comento sobre algún paciente en particular y comento para que no salga de aquí, que la persona no vaya a comentar con la gente de afuera. Que quede solo entre el equipo.* (ACS_05) ^||^ *Eso para mí no es ético; si entraste en el área como profesional entonces tienes que hacer lo que tu área o profesión te exige.* (TECENF_06) ^¶^ *Yo no puedo interferir en la conducta profesional de otra persona, del médico por ejemplo, porque cada uno tiene su propia reglamentación, cada uno tiene su legislación sobre lo que debe hacer. Entonces tenemos que trabajar dentro de los límites que nuestra profesión y actuación mandan.* (ENF_02)** **(-): no observado**
**Síntesis analítica:** El Clima Ético en el Entorno de Trabajo de la APS ^††^ se relaciona con la ética profesional/profesionalismo que, a su vez, se vincula tanto al cumplimiento de leyes y normas profesionales como a las normas y reglas institucionales.		
**Factor 2 – Benevolencia (BNV** ^‡‡^ **)**		
**ICE** ^†^ **– CUANTI** ^‡^	**Categor** í **a CUALI** ^§^	**Convergencias (+) o Divergencia (-)**
Se evidencia una importante valoración del: Bien (benevolencia) de todos los que trabajan en la unidad de salud (ítem 2); Bien (benevolencia) de las personas que trabajan en ella (ítem 1).	Coocurrencia concentrada de los ítems 2 y 1 con las categorías subjetivas “Percepciones y experiencias (construido en la interfaz entre impresiones positivas y negativas), y Pertenencia al grupo” (fuerza agregadora capaz de dar sentido al trabajo)	**(+):** *Respetándonos todos primeramente y tratando de hacer lo mejor que podemos dentro de la estructura, del entorno de trabajo, para el usuario.* (ENF_01)** *Nosotros somos una familia, procuramos respetar el sector de cada uno. En términos de organización, procuramos ser lo más organizados posible.* (AUXBUC_0) ^§§^ **(-): no observado**
**Síntesis analítica:** El Clima Ético en el Entorno de Trabajo de la APS ^††^ se relaciona con la benevolencia que, a su vez, se expresa en las percepciones y experiencias de los trabajadores de consideración por el bien común y de la unión entre profesionales, así como exige y promueve relaciones de mutuo respeto en el equipo.		

*PR = Principios y reglas; ^†^ICE = Inventario de Clima Ético; ^‡^CUANTI = Cuantitativo; ^§^CUALI = Cualitativo; ^||^ACS = Agente Comunitario de Salud (+ el orden de participación); ^¶^TECENF = Técnico de Enfermería (+ el orden de participación); ^**^ENF = Enfermero (+ el orden de participación); ^††^APS = Atención Primaria de la Salud; ^‡‡^BNV = Benevolencia; ^§§^AUXBUC = Auxiliar Bucal (+ el orden de participación)

En la Figura 4, se presenta el factor independencia e instrumentalismo (ítems 17 con 0,783; 16 con 0,783 y 18 con 0,716), al cual se integró el código cualitativo denominado agencia moral. Se observa convergencia cuando se manifiesta la capacidad y el empeño de los profesionales por guiarse por su propia ética personal mientras trabajan. Sin embargo, se observó una connotación complementaria atribuida a los sentidos de los discursos: por más que sean coherentes con el factor independencia, expresan también la influencia negativa sobre el CE por parte de sujetos negligentes, que desconocen o siguen poco los estándares éticos en el ET. La falta o negligencia se revela en los discursos como factor de insatisfacción en el servicio.

**Figura 4-:** Integración de resultados entre Factor (3) Independencia/instrumentalismo (IDP*) (QUANT^‡^) y categoría Ética personal (CUAL^§^), y Factor Benevolencia - Responsabilidad social (CUANT^‡^) y categoría Pertenencia al grupo y Clima Ético (CUAL^§^). Belém, PA, Brasil, 2021

Factor 3 – Independencia e instrumentalismo (IDP*)		
**ICE** ^†^ **– CUANTI** ^‡^	**Categor** í **a CUALI** ^§^	**Convergencias (+) o Divergencia (-)**
Se evidencia una importante valoración de: Guía de su propia ética personal (ítem 17); Por sí misma decidir lo que considera correcto e incorrecto (ítem 16); Protección de los propios intereses (ítem 18).	Coocurrencia concentrada de los ítems 17, 16 y 18 con las categorías éticas: “Agencia moral (capacidad de actuar para concretar acciones justas en el cuidado – ser sujeto moral), ética personal (valores y atributos personales que influyen en el actuar) y conflicto ético (disensos y enfrentamientos por valores y juicios morales)”.	*Escucho comentarios de los que procuro hasta huir. Solo se genera chisme cuando uno da cuerda. Hay gente que deja de trabajar y se queda hablando, escuchando.* (TEC_ENF_03) ^||^ *Contribuyo con mis actitudes; con el ejemplo se pueden modificar muchas cosas.* (MED_03) ^¶^ *Intento hacer mi parte ética con todas las personas, para ver si logramos tener un entorno ético.* (ACS_12)** *Las decisiones de la alta gerencia son un poco injustas y eso afecta nuestra relación en el trabajo.* (ACS_04)** *Pero hay situaciones en que uno no sabe cómo se trabaja y la persona viene y señala con el dedo juzgando.* (AUXBUC_03) ^††^ *Quedarse hablando de los datos de los pacientes, mantener conflicto entre colegas. Creo que ahí ya se pierde la ética.* (ENF_03) ^‡‡^ **(-): no observado**
**Síntesis analítica:** El Clima Ético en el Entorno de Trabajo de la APS ^§§^ se relaciona con la agencia moral, en la medida en que los sujetos ejercitan su juicio moral y definen elecciones razonables ante situaciones que involucran problemas o conflictos morales. Tales juicios recaen sobre hechos y circunstancias que, negativa (complemento que no representa una divergencia) o positivamente, remiten a aquellos principios y valores éticos que sirven de base autodeclarada (personales o profesionales).		
**Factor 4 – Sentido de comunidad/responsabilidad social (RS** ^||||^ **)**		
**ICE** ^†^ **– CUANTI** ^‡^	**Categor** í **a CUALI** ^§^	**Convergencias (+) o Divergencia (-)**
Se evidencia una importante valoración de la Preocupación por los intereses de los clientes y del público	Coocurrencia concentrada del ítem 14 con la categoría “subjetividad: Pertenencia al grupo; y ética: CE”.	**(+):** *Procuro atender a mi clientela y ayudo a mis colegas en lo que puedo; dentro de mis limitaciones, ayudo y contribuyo al trabajo.* (ENF_04) ^‡‡^ *Cuido mucho del servicio y voy más allá del servicio social, hago lo que puedo para que las cosas sucedan.* (ACS_01)** *No puedo ayudar tanto al paciente como me gustaría. Hay pacientes que no tienen condiciones de pagar exámenes, me los exigen y trato de hablar con la gerencia; a veces no logran programarlos, entonces me siento mal y eso limita mi trabajo.* (ACS_05)** **(-): no observado**
**Síntesis analítica:** El Clima Ético en el Entorno de Trabajo de la APS ^§§^ también se relaciona con el sentido de responsabilidad social de los trabajadores en el esfuerzo por realizar un trabajo digno, lo que incluye ítems objetivos constitutivos de un entorno de trabajo favorable.		

*IDP = Independencia e instrumentalismo; ^†^ICE = Inventario de Clima Ético; ^‡^CUANTI = Cuantitativo; ^§^CUALI = Cualitativo; ^||^TECENF = Técnico de Enfermería (+ el orden de participación); ^¶^MED = Medicina (+ el orden de participación); **ACS = Agente Comunitario de Salud (+ el orden de participación); ^††^AUXBUC = Auxiliar Bucal (+ el orden de participación); ^‡‡^ENF = Enfermero (+ el orden de participación); ^§§^APS = Atención Primaria de la Salud; ^||||^BAT-S = Síntomas secundarios

Aún en la Figura 4, se presenta el factor 4 - Responsabilidad Social (ítem 14 con 0,742), generado a partir de una subdivisión del factor Benevolencia (escala original). En la convergencia, el código esfuerzo expresa la preocupación activa de los profesionales por los intereses del público y por las personas usuarias de la APS; la crítica a las carencias de condiciones físicas y estructurales que escapan a la gobernabilidad y limitan la capacidad de actuación de los trabajadores, evidenciando la necesidad de la actuación de las políticas públicas en este ET.

## Discusión

### La diferenciación de los hallazgos según la ICE en portugués brasileño

Se observó que el instrumento utilizado para la medición del CE en los entornos de la APS se adapta conforme a algunas características de estos entornos; por lo tanto, no es absoluto. Los propios traductores de la versión brasileña señalan que se necesitan estudios futuros para identificar la adecuación de la estructura propuesta y su poder predictivo del CE en diferentes organizaciones^(15)^.

Los profesionales de la APS expresan niveles coherentes (estables) de CE en sus ET. Sin embargo, partiendo de los arquetipos de CE, donde se lo entiende como un atributo de una organización que puede ser captado por percepciones individuales^(15)^, fue posible identificar los siguientes factores dominantes en estos entornos: Principios y reglas; Benevolencia; Independencia (individualismo/instrumentalismo) y Responsabilidad social (desmembrado del factor Benevolencia).

Un hallazgo destacado del estudio fue la división del factor benevolencia, generando el nuevo factor de sentido de comunidad/responsabilidad social. Esto representa una contribución a futuras investigaciones, pues, aunque no expresa un conflicto con respecto al autor original de la escala, cuando sitúa el locus cosmopolita de la benevolencia^(32)^, ya en una percepción del CE orientada al sistema social y a los intereses colectivos. Sin embargo, al observarse una relevancia significativa del sentido de comunidad/responsabilidad social, hasta el punto de sugerir una discriminación mayor y constitutiva de un nuevo factor, puede vislumbrarse una posible insuficiencia de su abordaje como simple locus de benevolencia en el grupo estudiado.

Estudiar el CE en la APS requiere más análisis tanto cualitativos como cuantitativos del fenómeno, dado que el escenario está inmerso en dimensiones subjetivas y objetivas complejas y complementarias. Se entiende que la forma en que se atribuyen los significados y las comprensiones sobre la ética (su presencia y/o ausencia en el cotidiano del trabajo) y la forma en que la ética es “percibida y vivida” añade un sentido/valor al entorno de trabajo para cada profesional.

El factor 1 – “Principios y reglas” presentó la mayor carga factorial entre los profesionales. La afirmación que corresponde al Ítem 11 de la ICE – “Se espera que las personas cumplan, por encima de todo, las leyes y las normas profesionales” muestra, de forma general, que los profesionales comprenden y esperan el cumplimiento de las normas pertinentes de cada profesión, lo que entienden como elementos constitutivos del CE presentes en estos entornos.

Este hallazgo también fue semejante en la atención primaria, entre enfermeros y médicos que compartían la percepción de un clima de equipo favorable cuando hay respeto a los objetivos del equipo; y el reconocimiento de la enfermería como la categoría más cercana a los ideales del proceso de trabajo colectivo^(33)^.

La ética organizacional implica coherencia de las acciones con la misión, la visión y los valores de la organización, lo que hace necesarias formas de autorregulación dinámicas, valores compartidos entre diversos actores y atención a su cotidianeidad de interacciones sociales^(33)^. Los valores varían en los entornos de salud, incluso por las diferentes complejidades asistenciales o tecnológicas, y difieren de las reglas o normas constitutivas de la deontología de cada profesión^(34)^. Los valores y las normas profesionales deben considerarse en sus distinciones, pero también en la manera en que interactúan e integran la formación de identidades profesionales^(35)^.

Tal apropiación y sentido local otorgado a las normas sugiere que las intervenciones éticas en los entornos no necesitan estar limitadas a reglas y normas, sino ser estimuladas mediante la educación ética a los profesionales, de modo que puedan ejercer el juicio de manera honesta y responsable. La apertura al diálogo entre equipos puede hacer que el CE sea mejor comprendido y modificado por los profesionales.

En el factor 2 – “Benevolencia”, la afirmación “Nuestra mayor preocupación es el bien de todos los que trabajan en esta unidad de salud” (ítem 2 de la ICE) puede estar relacionada con el reconocimiento y la satisfacción con los gestores y con la adhesión del equipo a objetivos comunes. La cooperación entre profesionales y su involucramiento con las personas usuarias que acuden a la APS son elementos reportados como causas de satisfacción laboral de gestores en la estrategia de salud de la familia^(36)^. El hallazgo expresa cómo la unión de los profesionales y el sentido de equipo refuerzan el interés común y son ingredientes para el mantenimiento de un entorno de trabajo favorable.

La cooperación también se ve como un elemento de subversión del sufrimiento impuesto por las condiciones adversas presentes en los ET. El sentido de colectividad puede ser equivalente a la benevolencia en el sentido de contribuir a la construcción de objetivos comunes, a la comprensión mutua y al respeto a todos los que actúan bajo normas comunes^(37)^.

El factor 3 – “Independencia e individualismo” expresó el sentido de prioridad de aquello que es el interés propio dentro de determinada situación, o el interés en la acción determinado por lo que el individuo cree ser lo correcto. Fue posible detectar el grado de adhesión de los datos cualitativos al factor en el ítem “En este servicio, las personas se guían por su propia ética personal” (ítem 17 de la ICE).

El individualismo puede superponerse a los intereses del público o del equipo, contribuyendo a la sobrecarga de trabajo y al exceso de demanda a los colegas. El exceso de demandas propias y de terceros favorece la ocurrencia de efectos indeseados tanto sobre el entorno como sobre el público atendido, además de la insatisfacción por parte del profesional^(38)^. Lo mismo ocurre con el desgaste de las relaciones, en el que episodios conflictivos y éticamente negativos pueden provocar un clima desfavorable e incluso distrés moral^(39)^.

Obviamente, la insatisfacción, la sobrecarga de trabajo, el desgaste y el sufrimiento son fenómenos complejos, con múltiples causas y consecuencias, cabiendo aquí solo indicar la posibilidad de vinculación con procesos de agotamiento de la cooperación y estímulo a respuestas individualistas, de defensa de intereses personales o de autopreservación en tales ET.

El problema de acceso a oportunidades de educación ética para los trabajadores es una realidad en estos entornos. Para la categoría de los agentes comunitarios de salud (ACS), aún no existe un código de ética ni una entidad con atribución de fiscalización del ejercicio profesional, y mucho menos la percepción de ese respaldo ético de la categoría profesional ante situaciones recurrentes en la atención de personas usuarias de la APS. Tal vacío no impide que dichos trabajadores evalúen y formulen juicios morales sobre el entorno de acogida y asistencia^(40)^. De ahí la urgencia de inversiones y de la valorización de la educación permanente en salud (EPS) en la práctica concreta de los servicios de salud, con la inclusión del ACS en espacios de aprendizajes colectivos^(41)^.

Cuando no hay estímulos o acciones de EPS relacionados con situaciones que requieren competencia ética, las acciones/inacciones éticas pasan desapercibidas, omitidas, negligenciadas y, por lo tanto, sin resolutividad. Este aspecto puede traer sufrimiento moral a quien percibe tales lagunas; en esos casos, generalmente, el profesional de enfermería se sensibiliza para percibir problemas morales, pues su formación atraviesa tales definiciones y entendimientos^(42)^.

En el factor 4 – “Sentido de comunidad/responsabilidad social”, se aportó una contribución adicional del estudio mixto para un abordaje mutuamente complementario entre los factores 2 y 4. El factor 4 se presenta como un nuevo componente, derivado del segundo, no por divergencia, sino por profundización, pues una interpretación posible es que el sentido de comunidad/equipo o responsabilidad social se muestra como una forma de concretar el bien común (benevolencia – factor 2). Al mismo tiempo, una forma de benevolencia interna (entre pares) consolida e integra equipos de trabajo para que puedan ejercer mejor su papel social (el sentido de comunidad desdoblándose de la benevolencia). La responsabilidad social se configura como un criterio ético, no solo como locus de análisis o una característica cosmopolita del CE^(15)^, conforme la propuesta inicial de Victor y Cullen^(32)^. Así, se sugiere que el simple locus cosmopolita puede ser insuficiente para explicar los elementos aportados por el estudio mixto, pudiendo constituirse en un constructo propio.

La responsabilidad social se ve como un atributo del reconocimiento y pertenencia a un servicio y sistema público de salud: de la percepción de sí como apto para orientar, asistir y ayudar a personas que buscan atención. Es una noción apropiada por quienes trabajan con comunidades, nutren expectativas de resolutividad y enfrentan precariedades (estructurales, materiales y de seguridad) e impotencia en estos profesionales, pues extrapolan los límites de su unidad o campo de actuación^(37)^.

A pesar de la riqueza de los hallazgos, el estudio tuvo como limitación la ausencia de ampliación de la estratificación muestral de algunas ocupaciones que permitiera explorar diferencias con los factores implicados. Además, es preciso reconocer que el momento retratado estaba altamente impactado por la pandemia de COVID-19.

Estudios futuros con el objetivo de confirmar la efectividad de la ICE en otros entornos de la APS, en otras regiones del país, con aspectos diferenciados de lo encontrado en la ciudad del norte amazónico, podrán brindar nuevas perspectivas para tipologías del CE en los ET de salud. Aunque sin objetivo de estudio metodológico, la aplicación del instrumento permite sugerir que sea adaptado al escenario de la salud, incluyendo el factor responsabilidad social, y ajustes lingüísticos (por ejemplo: personas usuarias en lugar de clientes).

El estudio contribuye a una comprensión más profunda del concepto de CE y de su potencial para subsidiar la transformación en los ET, en especial en la APS. La captación de múltiples datos y visiones es útil para subsidiar iniciativas de perfeccionamiento de estos ET para sus trabajadores y personas usuarias. La calidad de los resultados del trabajo y de la oferta de cuidados no puede alcanzarse sin la debida atención a los trabajadores y a los entornos en los que actúan, incluidas las dimensiones éticas de dichos entornos. La contribución del estudio para la Enfermería se evidencia por ser estos los trabajadores grandemente afectados por sus ET y, también, por el papel de protagonismo que asumen para que sean transformados, siempre que la intersección entre ética y ET sea debidamente reconocida y problematizada. Además, el estudio mixto consiguió proyectar una configuración interpretativa ampliada de los resultados obtenidos con el inventario, apoyando la expresión de la complejidad del objeto, de modo que proporcione resultados indicativos de una futura adaptación del instrumento al campo de la salud.

## Conclusión

Varios factores relacionados con la medición del CE se mostraron pertinentes en los entornos de la APS analizados: la relevancia de los principios y reglas, cuando se refiere a la adhesión a las normas pertinentes a los códigos deontológicos de cada profesión; la benevolencia, al referir el bienestar de los sujetos que allí trabajan como orientador de acciones y elecciones; la independencia e instrumentalismo, que trae la discusión sobre los agentes morales y los conflictos que pueden surgir en el ET cuando la actitud ética es negligenciada o desconocida; y, por último, el sentido de comunidad/responsabilidad social, derivado de la benevolencia como preocupación por prestar un buen servicio público y favorecer el trabajo colectivo integrado.

La integración de los datos cuantitativos y cualitativos teniendo como punto de fusión los cuatro constructos/factores amplió y profundizó la comprensión sobre el objeto en síntesis analíticas que: refuerzan la importancia de la ética profesional/profesionalismo, anclada tanto en normas profesionales como institucionales; la fuerza de la benevolencia que se dirige tanto a la comunidad como a las relaciones entre profesionales en el equipo; el ejercicio de juicios morales imponiendo desafíos, pero también la imagen de agencia moral fundamental para que se perciban como sujetos morales de las acciones; y, por último, una importante distinción otorgada al sentido de comunidad (entre profesionales) y a la responsabilidad social, como constituyente de un CE en el escenario estudiado. Tal distinción del cuarto constructo fue una contribución importante y sustentada por datos cuantitativos y cualitativos, lo cual es deseable en aplicaciones de instrumentos validados, que requieren insumos para su perfeccionamiento.

El estudio trajo a discusión un escenario poco explorado en términos de CE: la APS y un municipio de la región amazónica. El objeto de estudio debe, necesariamente, considerar las particularidades de cada entorno; solo así tendrá un potencial transformador de escenarios, cualificando las experiencias de trabajo y sus resultados para personas y comunidades usuarias. Las dimensiones éticas de entornos y relaciones en la APS tienen en el CE una herramienta conceptual y analítica relevante, mientras que los estudios mixtos apoyan la comprensión de su complejidad.

## Data Availability

El conjunto de datos para este artículo está disponible em los siguientes enlaces: https://www.dropbox.com/scl/fi/ghsc4rw2f73k8bayct5om/Relat-rio_21072021_FATOR1.pdf?rlkey=q8iw7p7uqsuewvuneddefc721&st=yw2jjg7c&dl=0 https://www.dropbox.com/scl/fi/szyv293q7vss8xsf3oywi/Relat-rio_21072021_FATOR2.pdf?rlkey=9anfauf9xnr2ml54pcvjaz0r8&st=fasfir3h&dl=0 https://www.dropbox.com/scl/fi/kb5wrpm1ar7ycapkjk03p/Relat-rio_21072021_FATOR3.pdf?rlkey=pjrxfroumomx47ls5gbhlj7go&st=ppk2ee2r&dl=0 https://www.dropbox.com/scl/fi/ffxf9ybpp442gwea406o0/Relat-rio_21072021_FATOR4.pdf?rlkey=g5t0pa17v91wa81c4brl6aicl&st=x3vr3d00&dl=0 https://drive.google.com/file/d/1mApBFHq7PF6qADF9mzkAhq2KDyuCQJwN/view?usp=drive_link
